# Case Report: durable response to pulsed electric field ablation in combination with immune checkpoint inhibitors in HER2-low breast cancer through activation of interferon signaling

**DOI:** 10.3389/fonc.2026.1681010

**Published:** 2026-04-10

**Authors:** Yi Liu, Cynthia De La Garza-Ramos, Emmanuel Gabriel, Aziza Nassar, Beau Toskich, E. Aubrey Thompson, Saranya Chumsri

**Affiliations:** 1 Department of Quantitative Health Sciences, Mayo Clinic, Jacksonville, FL, United States; 2 Division of Interventional Radiology, Department of Radiology, Mayo Clinic, Jacksonville, FL, United States; 3 Department of Surgery, Mayo Clinic, Jacksonville, FL, United States; 4 Department of Laboratory Medicine and Pathology, Mayo Clinic, Jacksonville, FL, United States; 5 Department of Cancer Biology, Mayo Clinic, Jacksonville, FL, United States; 6 Department of Hematology-Oncology, Mayo Clinic, Jacksonville, FL, United States

**Keywords:** breast cancer, case report, HER-2 low, immune checkpoint inhibitor, interferon signaling, pulsed electric field, spatial transcriptomics

## Abstract

Pulsed Electric Field (PEF) ablation is a non-thermal cancer treatment that disrupts tumor cell membranes while preserving the immune microenvironment and promoting immunogenic cell death. We present a case of a patient with metastatic hormone receptor-positive, HER2-low breast cancer who achieved a durable clinical response after PEF in combination with an immune checkpoint inhibitor (pembrolizumab). The patient remained off systemic therapy for 14 months without disease progression. Single-cell spatial molecular imaging (SMI) and gene set enrichment analysis (GSEA) were performed to evaluate immune microenvironment changes pre- and post-PEF treatment. Spatial transcriptomics of the tumor cell compartment revealed a post-PEF enrichment of immune-related pathways, including interferon alpha/beta and gamma signaling and MHC class I antigen processing and presentation, alongside increased infiltration of immune cells: CD8+ T cells, plasmablasts, and monocytes within the tumor neighborhood. This hypothesis-generating case suggests PEF’s potential to reshape the tumor-immune microenvironment when combined with immune checkpoint blockade, warranting further studies on its role in combination immunotherapy strategies.

## 1 Introduction

Pulsed Electric Field (PEF) ablation is an emerging non-thermal approach for the local treatment of cancer that utilizes high-voltage electric pulses to induce tumor cell membrane disruption and apoptosis (1). Unlike conventional ablation methods, PEF has been shown to not only induce tumor cell apoptosis but also enhance anti-tumor immune responses while preserving the surrounding immune microenvironment. Recent studies suggest that PEF may promote immunogenic cell death, leading to the release of tumor antigens and danger-associated molecular patterns (DAMPs), which can stimulate dendritic cell activation and T-cell priming. Additionally, PEF appears to modulate the tumor microenvironment in a manner that favors immune infiltration and reduces immunosuppressive barriers (2). However, the precise mechanisms underlying these effects remain incompletely understood, warranting further investigation into its role in immune activation and potential synergies with immunotherapy. Here, we report a case of metastatic hormone receptor-positive, HER2-low breast cancer demonstrating a durable response following PEF ablation combined with pembrolizumab, with the patient remaining off systemic therapy for 14 months without disease progression. Tumor immune microenvironment alterations were characterized using single-cell spatial transcriptomics.

## 2 Case presentation

**Figure 1:**
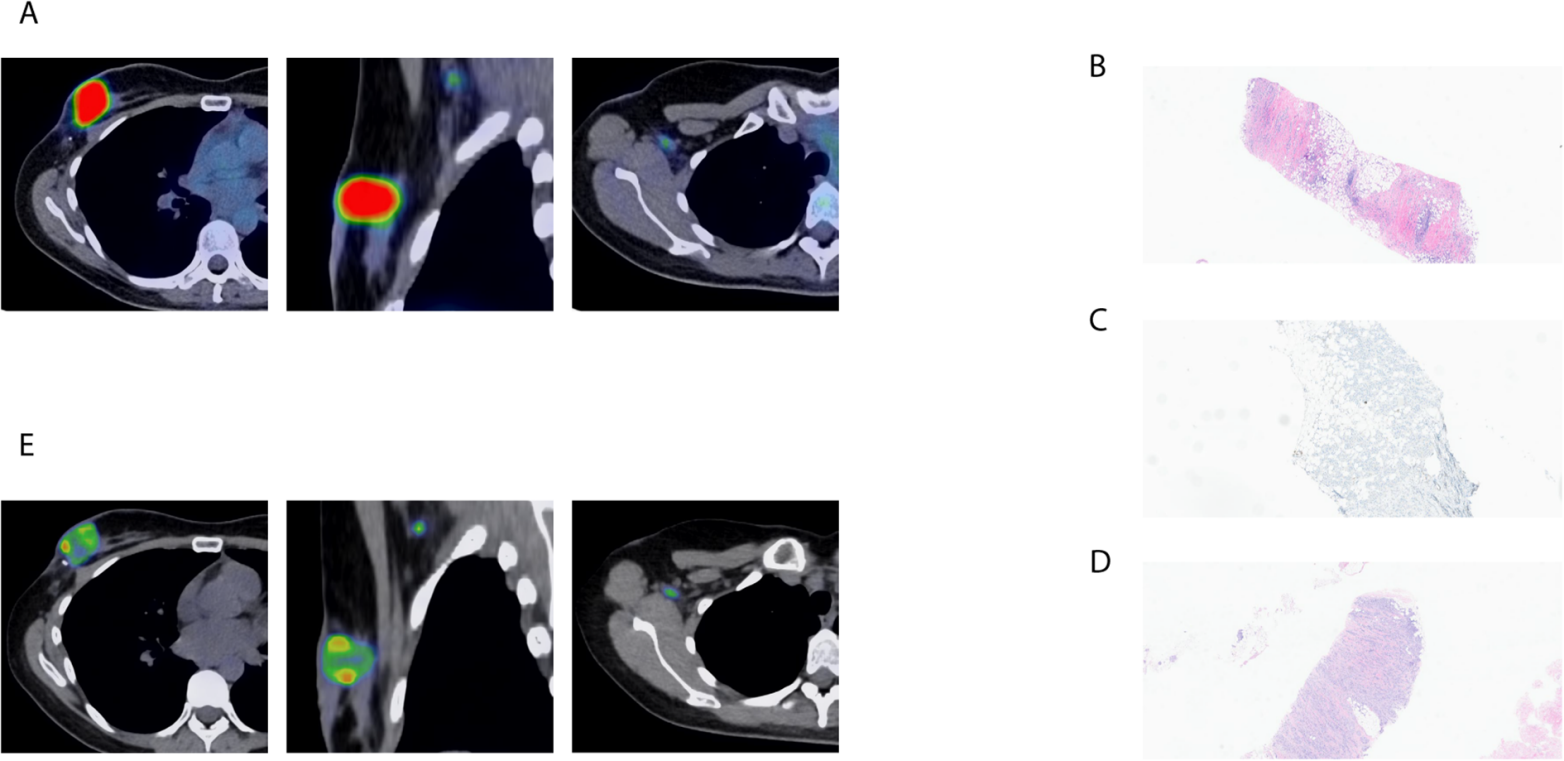
Imaging and histopathologic evaluation of isolated breast tumor progression before and after pulsed electric field (PEF) treatment. **(A)** Fluorodeoxyglucose (FDG) Positron Emission Tomography (PET) scan showing FDG avid isolated disease progression in the right breast. **(B)** The hematoxylin and eosin (H&E) staining of a core needle biopsy specimen prior to PEF showing grade 2 IDC, with low stromal tumor-infiltrating lymphocytes (sTILs) of 5%. **(C)** Programmed cell death ligand 1 (PD-L1) combined positive score (CPS) of 0. **(D)** The H&E staining of a repeated biopsy after PEF showing grade 2 IDC with low sTILs of 5%. **(E)** Follow up FDG PET scan 9 months after the PEF procedure showing smaller right axillary lymph node with stable right breast mass.

A 45-year-old patient presented to a tertiary care medical center for the management of the progression of breast cancer on capecitabine and trastuzumab. Patient’s original diagnosis was made five years prior with a large palpable mass in the right breast. Diagnostic evaluation revealed invasive ductal carcinoma (IDC) of the right breast with metastases to the ipsilateral axillary lymph nodes. Initial histopathologic evaluation revealed grade 2 IDC with estrogen receptor (ER)-positive (>95%), progesterone receptor (PR)-positive (15-20%), and human epidermal growth factor 2 (HER2)-low with immunohistochemistry (IHC) 2+ and fluorescence *in situ* hybridization (FISH) negative. The patient declined standard-of-care treatment and sought alternative therapies. The patient presented to our center three years after the original diagnosis with further progression of the disease, with a large fungating mass in the right breast eroding the skin, causing significant pain. Further workup with fludeoxyglucose (FDG) positron emission-tomography/computed tomography (PET/CT) scan showed that, unfortunately, the patient had already developed several bone metastases. The patient was initially started on ovarian suppression with leuprolide, tamoxifen, and abemaciclib. The patient had a repeated biopsy of the breast at our institution, which demonstrated grade 2 IDC, ER 76-100%, PR < 1%, and HER2 positive with IHC 3 +. Given the HER2 conversion, treatment was switched to capecitabine, trastuzumab, and pertuzumab, similar to the phase III trial reported by Urruticoechea et al. (3). The patient did well with this treatment for 21 months but developed isolated disease progression in the right breast (**Figure 1A**). Following a multidisciplinary discussion, the decision was to proceed with local therapy for the breast lesion. However, the patient declined standard-of-care treatment and received alternative therapy including tamoxifen at an outside facility. At the time, the patient was referred to the Interventional Radiology team for the consideration of local therapy. Patient was ineligible for cryoablation due to the extent of local disease and proximity to the skin surface, but patient was felt to be a candidate for percutaneous PEF ablation. Since the isolated breast progression, pembrolizumab was added 5 days prior to PEF ablation, with the hypothesis that PEF-induced immunogenic cell death could enhance antitumor immune responses. This approach was not based on PD-L1 status or other established predictive biomarkers for immune checkpoint inhibitor response.

On the day of ablation, a 14-gauge core biopsy of the tumor was obtained first, and the samples were evaluated by a cytotechnologist to confirm malignant cells. The 19-gauge PEF needle was then advanced to the same area under ultrasound guidance. PEF ablation was performed under cardiac gating for 100 pulses utilizing the Galvanize Aliya™ System and the percutaneous Aliya Ablation Device (Galvanize Therapeutics, San Carlos, CA). Detailed pulse parameters (amplitude, width, frequency) and electrode specifications followed the manufacturer’s standard protocol; granular dosimetry data were not systematically recorded.

The PEF needle was subsequently advanced to three additional separate locations in the mid and caudal moieties of the tumor, and 100 pulses were delivered at each station. The pre-PEF biopsy demonstrated grade 2 invasive ductal carcinoma (**Figure 1B**), ER 76–100%, PR 76–100%, HER2 IHC 2+ with FISH negative (ratio 1.27; copy number 3.1), consistent with HER2-low status and indicating a shift from the prior HER2-positive (IHC 3+) result; Ki67 was 12.8%. This specimen was noted to have low stromal tumor-infiltrating lymphocytes (sTILs) of 5% with programmed cell death ligand 1 (PD-L1) combined positive score (CPS) of 0 (**Figure 1C**). Follow-up FDG PET/CT three weeks after ablation demonstrated reduced FDG uptake in the right breast carcinoma and axillary lymphadenopathy The patient continued three more cycles of pembrolizumab, capecitabine, and trastuzumab over approximately two months before pembrolizumab was discontinued due to immune-related hypothyroidism. Magnetic resonance imaging (MRI) performed two months after ablation was suggestive of disease progression in the right breast with increased size of the dominant mass upper outer right breast as well as surrounding non-mass enhancement extending into the medial and inferior breast. The patient underwent repeated biopsy 3.8 months after the initial PEF to rule out pseudoprogression. The repeat biopsy revealed grade 2 invasive ductal carcinoma (**Figure 1D**), ER 76–100%, PR 11–50%, and HER2 IHC 2+ with FISH negative (ratio 1.24; copy number 3.2), again consistent with HER2-low status; Ki67 was 19.1%. This specimen was also noted to have low sTILs at 5%, and PD-L1 was negative with a CPS of 0. Based on the results of this biopsied specimen, it was felt that the patient had disease progression and was recommended to change treatment. Following pembrolizumab discontinuation, the patient developed persistent fatigue attributed to immune-related hypothyroidism and declined further systemic therapy.

Nevertheless, the patient continued follow-up and returned for follow-up scans. Nine months after the PEF procedure, the follow-up FDG PET/CT showed that the right axillary lymph node continued to decrease in size (**Figure 1E**), and the breast mass was stable. The patient continued to be off systemic therapy without disease progression for a total of 14 months until the follow-up FDG PET/CT scan started to show mild disease progression with two new bone metastases (**Table 1**). Following disease progression at 14 months, the patient was restarted on systemic therapy with exemestane, leuprolide, trastuzumab, and pertuzumab.

**Table 1:** Clinical response assessment using RECIST 1.1 and irRECIST criteria.

Days	Breast lesion size (cm)	Breast SUV	LN lesion size (cm)	LN SUV	% Change	RECIST 1.1	irRECIST
Day -98	3.2 x 2.6	8.2	1.6 x 0.7	2.5	—	Baseline	Baseline
PEF	—	—	—	—	—	—	—
Day +3	3.0 x 1.6	4.6	1.6 x 0.6	2.3	-4.2%	Stable disease	Stable disease
Day +156	3.2 x 2.4	10.7	2.3 x 1.2	10.3	+14.6%	Stable disease	Stable disease
Day +280	3.2 x 2.4	14.3	1.7 x 0.8	14.4	+2.1%	Stable disease	Stable disease
Day +455	3.8 x 2.8	17.3	2.1 x 0.7	17.3	+28.3%	Progression with new lesions	Progression with new lesions

PEF, pulsed electric field; SUV, standardized uptake value; LN, lymph node; RECIST, Response Evaluation Criteria in Solid Tumors; irRECIST, immune-related RECIST. Day 0 = PEF procedure date. % Change calculated from sum of longest diameters relative to baseline.

**Figure 2:**
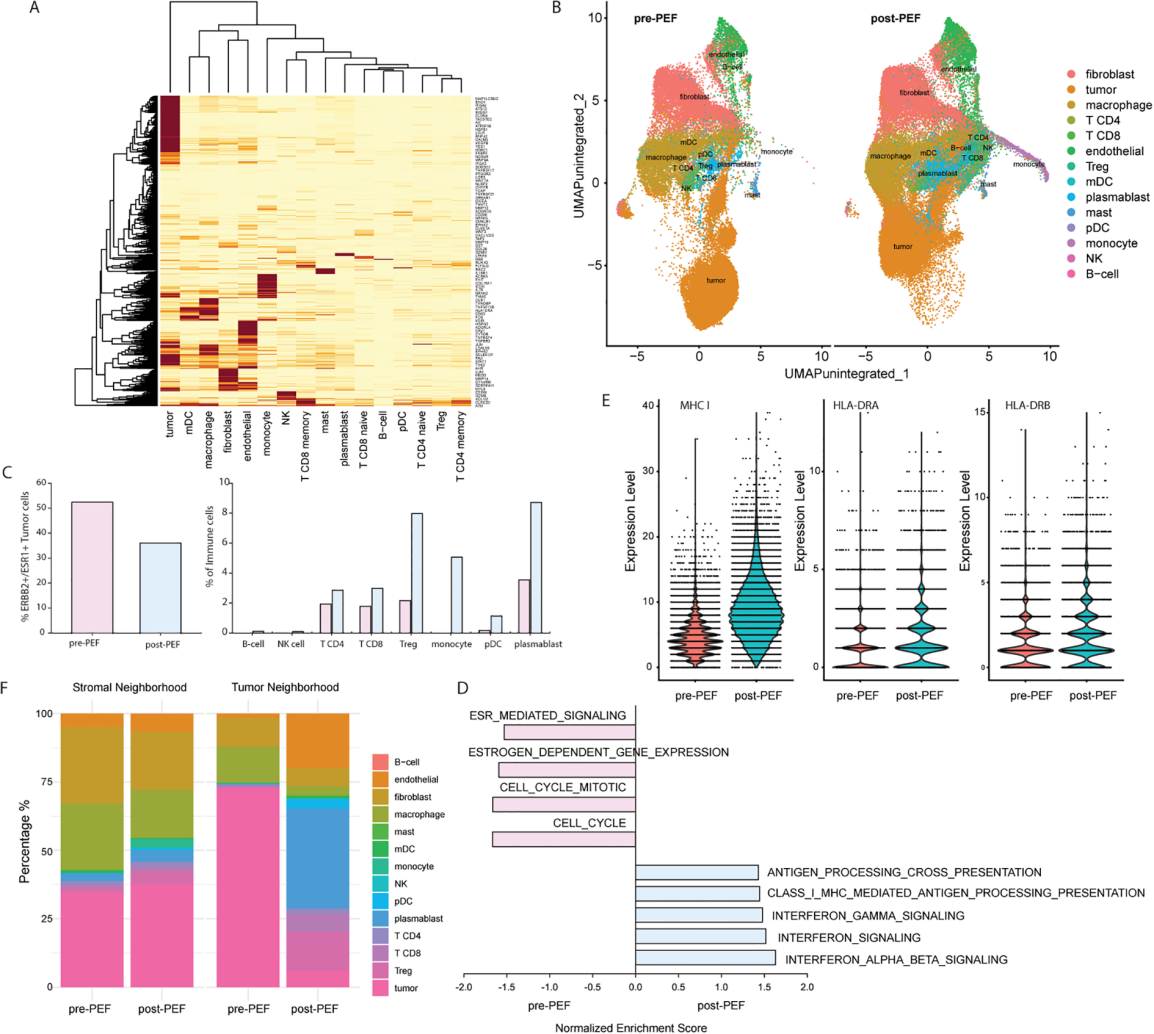
Single-cell spatial profiling reveals cellular and molecular changes in the tumor microenvironment before and after PEF treatment. **(A)** Heatmap of cell type identification across single-cell CosMx™ SMI data. **(B)** UMAP visualization shows major cell types before (pink) and after (blue) PEF treatment. **(C)** Distribution of tumor cells (left) and major immune cell types (right) before and after PEF treatment. **(D)** Gene set enrichment analysis (GSEA) of significantly enriched REACTOME (p ≤ 0.05, FDR < 0.25) comparing pre- and post-PEF samples. **(E)** Expression of MHC I (left), HLA-DRA (middle) and HLA-DRB (right) in tumor cells before and after PEF treatment. **(F)** Spatial distribution of tumor and 13 non-tumor cell types across two distinct spatial neighborhoods before and after PEF treatment.

We further used the NanoString CosMx™ Spatial Molecular Imaging (SMI) platform to evaluate 960 transcripts (Universal Cell Characterization 1K Panel, Supplementary table) single-cell spatial analysis to determine cell type composition between two samples. Semi-supervised cell typing identified 14 distinct cell types: B cells, endothelial cells, fibroblasts, macrophages, mast cells, myeloid dendritic cells (mDCs), monocytes, natural killer (NK) cells, plasmacytoid dendritic cells (pDCs), plasmablasts, T CD4 cells (T CD4 memory and T CD4 naïve), T CD8 cells (T CD8 memory and T CD8 naïve), T regulatory cells (Tregs), and tumor cells. Expression patterns of key cell type markers are shown in **Figure 2A**. A UMAP representation of the transcriptional profiles from both samples is also shown in **Figure 2B**. Comparing the distribution of non-tumor cell types between the pre- and post-PEF samples, several immune cell populations, including macrophages (39.73% vs. 26.50%), mDCs (0.95% vs. 0.35%), and mast cells (0.72% vs. 0.42%), showed a decrease in the post-PEF sample (**Table 1**). In contrast, other immune cell types exhibited marked increases in the post-PEF sample, including plasmablasts (3.56% vs. 8.73%), monocytes (0.02% vs. 5.08%), B cells (0.004% vs. 0.14%), CD4 T cells (1.96% vs. 2.88%), CD8 T cells (1.81% vs. 3.00%), regulatory T cells (Tregs 2.19% vs. 7.98%), plasmacytoid dendritic cells (pDCs 0.19% vs. 1.16%), and natural killer (NK) cells (0.02% vs. 0.12%) (**Figure 2C** and **Table 1**).

UMAP analysis revealed distinct distributions of tumor cells between the pre- and post-PEF samples (**Figure 2B**), suggesting potential differences in biological processes between the two conditions. To explore these differences, we performed a REACTOME pathway analysis on the combined tumor cell transcriptomes. Gene set enrichment analysis (GSEA) indicated that the pre-PEF sample was significantly enriched in pathways related to the cell cycle and ESR-related gene expression and signaling (p ≤ 0.05, FDR < 0.25, **Figure 2D**). In contrast, the post-PEF sample demonstrated significant enrichment in Interferon Alpha (IFNα), Beta (IFNβ), and Gamma (IFNγ) signaling as well as major histocompatibility complex (MHC) class I antigen processing and presentation pathways (**Figure 2D**), indicating a shift in immune-related processes after treatment. We also noted increased expressions of both MHC class I and II components (MHC I, HLA-DRA, and HLA-DRB) in the post-PEF samples (**Figure 2E**).

Despite the discrepancy between sTIL quantification, which indicated stable sTIL levels, and SMI results, which revealed increased immune infiltration, we further analyzed the cellular composition distribution across two distinct spatial neighborhoods. The tumor neighborhood (TN) was defined as all cells located within 50 microns of a tumor cell, and the stroma neighborhood (SN) included all cells that were outside this 50-micron boundary. The distribution of cell types in TN and SN is summarized in **Table 2**. Consistent with the sTIL score, which reflects immune infiltrate within the stroma, the proportion of non-tumor immune cells in SN remained relatively stable between pre- and post-PEF samples (65.5% vs. 62.4%, **Figure 2F**). However, within TN, tumor cells were markedly decreased in the post-PEF sample (5.9% vs. 72.8%), but substantial increases in non-tumor immune cells, including pDCs (0.05% vs. 3.5%), plasmablasts (0.33% vs. 36.6%), CD4 T cells (0.5% vs. 1.9%), CD8 T cells (0.35% vs. 6.8%), and Tregs (0.43% vs. 14.2%, **Figure 2F**) (**Table 3**). These findings highlight a dynamic remodeling of the TN, characterized by a marked reduction in tumor cells and a concurrent enrichment of immune cell infiltrates within TN following PEF treatment.

**Table 2:** Immune cell composition in pre- and post-PEF samples.

Tissue	Cell type	Freq	Percentage (%)
pre-PEF	B-cell	1	0
post-PEF	B-cell	60	0.14
pre-PEF	NK	4	0.02
post-PEF	NK	55	0.12
pre-PEF	T CD4	460	1.96
post-PEF	T CD4	1269	2.88
pre-PEF	T CD8	425	1.81
post-PEF	T CD8	1324	3
pre-PEF	Treg	514	2.19
post-PEF	Treg	3519	7.98
pre-PEF	endothelial	1625	6.91
post-PEF	endothelial	4930	11.19
pre-PEF	fibroblast	9862	41.94
post-PEF	fibroblast	14300	32.44
pre-PEF	mDC	224	0.95
post-PEF	mDC	156	0.35
pre-PEF	macrophage	9342	39.73
post-PEF	macrophage	11680	26.5
pre-PEF	mast	169	0.72
post-PEF	mast	184	0.42
pre-PEF	monocyte	4	0.02
post-PEF	monocyte	2239	5.08
pre-PEF	pDC	45	0.19
post-PEF	pDC	512	1.16
pre-PEF	plasmablast	838	3.56
post-PEF	plasmablast	3848	8.73

**Table 3:** Cellular composition of spatial neighborhoods in pre- and post-PEF samples.

Tissue	Cell type	Neighborhood	Freq	Percentage
pre-PEF	B-cell	stromal neighborhood	1	0
post-PEF	B-cell	stromal neighborhood	59	0.09
pre-PEF	endothelial	stromal neighborhood	1271	4.86
post-PEF	endothelial	stromal neighborhood	4358	6.57
pre-PEF	fibroblast	stromal neighborhood	7352	28.13
post-PEF	fibroblast	stromal neighborhood	14110	21.28
pre-PEF	macrophage	stromal neighborhood	6279	24.02
post-PEF	macrophage	stromal neighborhood	11581	17.47
pre-PEF	mast	stromal neighborhood	135	0.52
post-PEF	mast	stromal neighborhood	180	0.27
pre-PEF	mDC	stromal neighborhood	182	0.7
post-PEF	mDC	stromal neighborhood	132	0.2
pre-PEF	monocyte	stromal neighborhood	3	0.01
post-PEF	monocyte	stromal neighborhood	2236	3.37
pre-PEF	NK	stromal neighborhood	4	0.02
post-PEF	NK	stromal neighborhood	51	0.08
pre-PEF	pDC	stromal neighborhood	33	0.13
post-PEF	pDC	stromal neighborhood	412	0.62
pre-PEF	plasmablast	stromal neighborhood	760	2.91
post-PEF	plasmablast	stromal neighborhood	2796	4.22
pre-PEF	TCD4	stromal neighborhood	344	1.32
post-PEF	TCD4	stromal neighborhood	1215	1.83
pre-PEF	TCD8	stromal neighborhood	342	1.31
post-PEF	TCD8	stromal neighborhood	1130	1.7
pre-PEF	Treg	stromal neighborhood	413	1.58
post-PEF	Treg	stromal neighborhood	3111	4.69
pre-PEF	tumor	stromal neighborhood	9020	34.51
post-PEF	tumor	stromal neighborhood	24924	37.6
pre-PEF	B-cell	tumor neighborhood	0	0
post-PEF	B-cell	tumor neighborhood	1	0.03
pre-PEF	endothelial	tumor neighborhood	354	1.51
post-PEF	endothelial	tumor neighborhood	572	19.9
pre-PEF	fibroblast	tumor neighborhood	2510	10.69
post-PEF	fibroblast	tumor neighborhood	190	6.61
pre-PEF	macrophage	tumor neighborhood	3063	13.05
post-PEF	macrophage	tumor neighborhood	99	3.44
pre-PEF	mast	tumor neighborhood	34	0.14
post-PEF	mast	tumor neighborhood	4	0.14
pre-PEF	mDC	tumor neighborhood	24	0.18
post-PEF	mDC	tumor neighborhood	2	0.83
pre-PEF	monocyte	tumor neighborhood	1	0
post-PEF	monocyte	tumor neighborhood	3	0.1
pre-PEF	NK	tumor neighborhood	0	0
post-PEF	NK	tumor neighborhood	4	0.14
pre-PEF	pDC	tumor neighborhood	12	0.05
post-PEF	pDC	tumor neighborhood	100	3.48
pre-PEF	plasmablast	tumor neighborhood	78	0.33
post-PEF	plasmablast	tumor neighborhood	1052	36.59
pre-PEF	TCD4	tumor neighborhood	116	0.49
post-PEF	TCD4	tumor neighborhood	54	1.88
pre-PEF	TCD8	tumor neighborhood	83	0.35
post-PEF	TCD8	tumor neighborhood	194	6.75
pre-PEF	Treg	tumor neighborhood	101	0.43
post-PEF	Treg	tumor neighborhood	408	14.19
pre-PEF	tumor	tumor neighborhood	17083	72.76
post-PEF	tumor	tumor neighborhood	170	5.91

## 3 Discussion

Immune checkpoint inhibitors (ICIs) have emerged as effective cancer therapies in multiple types of tumors. ICIs exert their activity mainly through enhancing pre-existing host immune responses. However, studies have demonstrated low clinical activity of ICI in immunologically cold tumors, particularly in hormone receptor-positive HER2-negative breast cancer in the metastatic setting. In the previous phase Ib JAVELIN trial of anti-PD-L1 avelumab, the objective response rate (ORR) of single-agent avelumab was merely 2.8% (4). Even in HER2-positive breast cancer, the combination of a checkpoint inhibitor with anti-HER2 therapy only demonstrated a marginal benefit. In the single-arm multicenter phase IB/II PANACEA trial (5), the objective response rate was 15% in 40 patients with PD-L1-positive tumors. However, no objective response was observed among any of the 12 patients with PD-L1-negative tumors. Notably, this patient’s tumor exhibited HER2 receptor conversion: initially HER2-low (IHC 2+/FISH-negative) at original diagnosis, converting to HER2-positive (IHC 3+) on repeat biopsy three years later, and reverting to HER2-low (IHC 2+/FISH-negative) on subsequent biopsies. Due to potential tumor heterogeneity with HER2 expression, anti-HER2 therapy was continued in this patient’s case.

Considering the patient’s hormone receptor–positive, HER2-low, PD-L1–negative disease (CPS 0) and prior progression on capecitabine and trastuzumab, the brief two-month exposure to pembrolizumab before it was discontinued for toxicity makes it unlikely that pembrolizumab alone accounted for the 14-month period of disease stability observed off systemic therapy.

In addition, this case also highlighted the importance of the spatial distribution of immune infiltrate. When evaluated using conventional pathology quantification of sTILs on hematoxylin and eosin (H&E) staining, there was no significant increase in sTILs observed. This observation led to an initial impression of disease progression. However, further evaluation with spatial single-cell analysis using CosMx technology revealed increases in overall immune infiltration. We then further evaluated immune infiltration within the tumor versus stromal compartments (6–10). Similar to what was observed with TIL in the stroma, immune cell infiltration quantified by CosMx within the stromal compartment was relatively stable. In contrast, there were increases in several immune infiltrates within the tumor compartment. Our case report underscores the critical importance of assessing not only the overall quantity but also the spatial distribution of immune cells in the tumor microenvironment. Furthermore, our findings suggest that PEF may exert its antitumor effects through modulation of the tumor-immune microenvironment, notably via activation of the interferon response pathways and upregulation of MHC expression in tumor cells.

These descriptive findings suggest that PEF ablation, in combination with pembrolizumab may have contributed to immune remodeling within the tumor microenvironment. Similar findings were reported in both pre-clinical (11, 12) and clinical settings (13, 14). In summary, this hypothesis-generating case report describes the clinical course and detailed spatial transcriptomic landscape of a patient who experienced a prolonged clinical benefit following PEF ablation combined with pembrolizumab. While the observed immune remodeling is compelling, prospective clinical trials incorporating longitudinal biomarker assessments and serial biopsies are essential to establish whether PEF can consistently enhance or sustain the efficacy of immune checkpoint inhibitors. 
