# Supplementary material for: Case Report: durable response to pulsed electric field ablation in combination with immune checkpoint inhibitors in HER2-low breast cancer through activation of interferon signaling
Source: Front Oncol. 2026 Apr 10;16:1681010. doi: 10.3389/fonc.2026.1681010 (PMC13105998; doi:10.3389/fonc.2026.1681010)
Supplement: Supplementary file 1 [file DataSheet1.pdf]

Supplementary Table: CosMx™ Universal Cell Characterization Panel

Gene Symbol	Alias(es)	Gene Name(s)
AATK	lemur tyrosine kinase 1,protein phosphatase 1, regulatory subunit 77; AATYK,KIAA0641,LMTK1,LMR1,AATYK1,PPP1R77	apoptosis associated tyrosine kinase
ABL1	JTK7,c-ABL,p150; v-abl Abelson murine leukemia viral oncogene homolog 1,c-abl oncogene 1, receptor tyrosine kinase,c-abl oncogene 1, non-receptor tyrosine kinase	ABL proto-oncogene 1, non-receptor tyrosine kinase
ABL2	ARG; Abelson-related gene; v-abl Abelson murine leukemia viral oncogene homolog 2 (arg, Abelson-related gene),v-abl Abelson murine leukemia viral oncogene homolog 2,c-abl oncogene 2, non-receptor tyrosine kinase	ABL proto-oncogene 2, non-receptor tyrosine kinase
ACE	ACE1,CD143; angiotensin I converting enzyme (peptidyl-dipeptidase A) 1; peptidyl-dipeptidase A	angiotensin I converting enzyme
ACE2	ACEH; angiotensin I converting enzyme (peptidyl-dipeptidase A) 2,angiotensin I converting enzyme 2; peptidyl-dipeptidase A	angiotensin converting enzyme 2
ACKR1	atypical chemokine receptor 1,glycoprotein D; CCBP1,GPD,Dfy,CD234; Duffy blood group,Duffy blood group, chemokine receptor,Duffy blood group, atypical chemokine receptor	atypical chemokine receptor 1 (Duffy blood group)
ACKR3	chemokine orphan receptor 1,chemokine (C-X-C motif) receptor 7; RDC1,GPR159	atypical chemokine receptor 3
ACKR4	chemokine (C-C motif) receptor-like 1; CCR11,CCBP2,VSHK1,CCX-CKR,PPR1	atypical chemokine receptor 4
ACTA2	ACTSA; actin, alpha 2, smooth muscle, aorta	actin alpha 2, smooth muscle
ACTG2	ACTSG; actin, gamma 2, smooth muscle, enteric	actin gamma 2, smooth muscle
ACVR1	activin A receptor, type I; SKR1,ALK2,ACVR1A	activin A receptor type 1
ACVR1B	ALK4,SKR2,ActRIB; activin A receptor, type IB	activin A receptor type 1B
ACVR2A	activin A receptor, type II,activin A receptor, type IIA; ACTRII	activin A receptor type 2A
ACVRL1	HHT2,ALK1,HHT; activin A receptor type II-like 1,activin A receptor type IL; activin receptor-like kinase 1	activin A receptor like type 1
ADGRA2	TEM5,DKFZp434C211,DKFZp434J0911,KIAA1531,FLJ14390; tumor endothelial marker 5; G protein-coupled receptor 124	adhesion G protein-coupled receptor A2
ADGRA3	G protein-coupled receptor 125; FLJ38547,PGR21	adhesion G protein-coupled receptor A3
ADGRB2	brain-specific angiogenesis inhibitor 2	adhesion G protein-coupled receptor B2
ADGRB3	brain-specific angiogenesis inhibitor 3; KIAA0550	adhesion G protein-coupled receptor B3
ADGRD1	DKFZp434B1272,PGR25; G protein-coupled receptor 133	adhesion G protein-coupled receptor D1
ADGRE1	egf-like module containing, mucin-like, hormone receptor-like sequence 1,egf-like module containing, mucin-like, hormone receptor-like 1	adhesion G protein-coupled receptor E1
ADGRE2	egf-like module containing, mucin-like, hormone receptor-like sequence 2,egf-like module containing, mucin-like, hormone receptor-like 2; CD312	adhesion G protein-coupled receptor E2
ADGRE5	TM7LN1; leukocyte antigen CD97,seven-span transmembrane protein,seven-transmembrane, heterodimeric receptor associated with inflammation,seven transmembrane helix receptor; CD97 antigen,CD97 molecule	adhesion G protein-coupled receptor E5
ADGRF1	hGPCR36,PGR19; G protein-coupled receptor 110	adhesion G protein-coupled receptor F1
ADGRF3	G protein-coupled receptor 113; hGPCR37,PGR23	adhesion G protein-coupled receptor F3
ADGRF4	FLJ38076,PGR18; G protein-coupled receptor 115	adhesion G protein-coupled receptor F4
ADGRF5	DKFZp564O1923,KIAA0758; G protein-coupled receptor 116	adhesion G protein-coupled receptor F5
ADGRG1	G protein-coupled receptor 56; TM7LN4,TM7XN1	adhesion G protein-coupled receptor G1
ADGRG2	G protein-coupled receptor 64; HE6,TM7LN2,EDDM6; epididymal protein 6	adhesion G protein-coupled receptor G2
ADGRG3	Pb99,PGR26; G protein-coupled receptor 97	adhesion G protein-coupled receptor G3
ADGRG5	G protein-coupled receptor 114; PGR27	adhesion G protein-coupled receptor G5
ADGRG6	G protein-coupled receptor 126; FLJ14937	adhesion G protein-coupled receptor G6
ADGRL1	latrophilin 1; KIAA0821,CIRL1,LEC2	adhesion G protein-coupled receptor L1
ADGRL2	latrophilin 1,latrophilin 2; calcium-independent alpha-latrotoxin receptor 2; KIAA0786,LEC1,CIRL2	adhesion G protein-coupled receptor L2
ADGRL4	EGF, latrophilin and seven transmembrane domain containing 1; ETL	adhesion G protein-coupled receptor L4

ADGRV1	monogenic, audiogenic seizure susceptibility 1 homolog (mouse),G protein-coupled receptor 98; DKFZp761P0710,KIAA0686,FEB4,VLGR1	adhesion G protein-coupled receptor V1
ADIPOQ	ACRP30,apM1,GBP28,adiponectin; adipocyte, C1Q and collagen domain containing; adipose most abundant gene transcript 1,adiponectin precursor	adiponectin, C1Q and collagen domain containing
ADIRF	chromosome 10 open reading frame 116; APM2,AFRO; adipose specific 2,adipose most abundant gene transcript 2,adipogenesis factor rich in obesity	adipogenesis regulatory factor
ADM2	AM2,FLJ21135	adrenomedullin 2
ADORA2A	RDC8	adenosine A2a receptor
AGR2	protein disulfide isomerase family A, member 17; anterior gradient 2 homolog (Xenopus laevis); XAG-2,HAG-2,AG2,PDIA17	anterior gradient 2, protein disulphide isomerase family member
AHI1	FLJ20069,ORF1,JBTS3; Jouberin; Abelson helper integration site	Abelson helper integration site 1
AHR	bHLHe76	aryl hydrocarbon receptor
AKT1	protein kinase B; RAC,PKB,PRKBA,AKT,RAC-alpha; v-akt murine thymoma viral oncogene homolog 1	AKT serine/threonine kinase 1
ALCAM	CD166,MEMD; activated leucocyte cell adhesion molecule	activated leukocyte cell adhesion molecule
ANGPT1	KIAA0003,Ang1	angiopoietin 1
ANGPT2	Ang2	angiopoietin 2
ANGPT4		angiopoietin 4
ANGPTL1	angioarrestin; angiopoietin-like 1; ANG3,AngY,ARP1	angiopoietin like 1
ANXA1		annexin A1
ANXA2	LIP2; annexin II	annexin A2
ANXA4		annexin A4
APOA1	apolipoprotein A-I	apolipoprotein A1
APOB	apolipoprotein B (including Ag(x) antigen)	apolipoprotein B
APOD		apolipoprotein D
APP	peptidase nexin-II; Alzheimer disease,amyloid beta (A4) precursor protein; alpha-sAPP	amyloid beta precursor protein
AQP3	GIL; aquaporin 3,aquaporin 3 (GIL blood group); Gill blood group	aquaporin 3 (Gill blood group)
AR	AIS,NR3C4,SMAX1,HUMARA; dihydrotestosterone receptor,spinal and bulbar muscular atrophy; testicular feminization,Kennedy disease	androgen receptor
AREG	schwannoma-derived growth factor,amphiregulin B	amphiregulin
ARF1		ADP ribosylation factor 1
ARG1	arginase, liver	arginase 1
ARHGDIB	Ly-GDI,RhoGDI2; Rho GDP dissociation inhibitor (GDI) beta	Rho GDP dissociation inhibitor beta
ARTN	NBN,EVN,ENOVIN; neublastin,neurotrophic factor	artemin
ATF3		activating transcription factor 3
ATG10	APG10 autophagy 10-like (S. cerevisiae),ATG10 autophagy related 10 homolog (S. cerevisiae); DKFZP586I0418,FLJ13954	autophagy related 10
ATG12	APG12; APG12 autophagy 12-like; Apg12 (autophagy 12, S. cerevisiae)-like,APG12 autophagy 12-like (S. cerevisiae),ATG12 autophagy related 12 homolog (S. cerevisiae)	autophagy related 12
ATG5	APG5 (autophagy 5, S. cerevisiae)-like,APG5 autophagy 5-like (S. cerevisiae),ATG5 autophagy related 5 homolog (S. cerevisiae); ASP,APG5,hAPG5	autophagy related 5
ATM	TEL1, telomere maintenance 1, homolog (S. cerevisiae); ataxia telangiectasia mutated (includes complementation groups A, C and D),ataxia telangiectasia mutated; TEL1,TELO1	ATM serine/threonine kinase
ATR	ataxia telangiectasia and Rad3 related; FRP1,SCKL,SCKL1,MEC1; MEC1, mitosis entry checkpoint 1, homolog (S. cerevisiae)	ATR serine/threonine kinase
AXL	UFO,JTK11,Tyro7,ARK	AXL receptor tyrosine kinase
AZGP1	ZA2G,ZAG; alpha-2-glycoprotein 1, zinc	alpha-2-glycoprotein 1, zinc-binding
AZU1	AZU,CAP37,AZAMP,HBP,NAZC,HUMAZUR; cationic antimicrobial protein 37,heparin-binding protein,neutrophil azurocidin	azurocidin 1
B2M		beta-2-microglobulin

B3GNT7	beta3GnT7	UDP-GlcNAc:betaGal beta-1,3-N-acetylglucosaminyltransferase 7
BAG3	BCL2 associated athanogene 3; BAG family molecular chaperone regulator 3	BAG cochaperone 3
BATF3	Jun dimerization protein 1; basic leucine zipper transcription factor, ATF-like 3; JUNDM1,SNFT,JDP1	basic leucine zipper ATF-like transcription factor 3
BAX	BCL2-associated X protein,BCL2 associated X protein; BCL2L4	BCL2 associated X, apoptosis regulator
BCL2	B-cell CLL/lymphoma 2,BCL2, apoptosis regulator; protein phosphatase 1, regulatory subunit 50; Bcl-2,PPP1R50	BCL2 apoptosis regulator
BCL2L1	BCLX,BCL2L,Bcl-X,bcl-xL,bcl-xS,PPP1R52; protein phosphatase 1, regulatory subunit 52	BCL2 like 1
BECN1	ATG6 autophagy related 6 homolog (S. cerevisiae); beclin 1 (coiled-coil, moesin-like BCL2 interacting protein),beclin 1, autophagy related; ATG6,VPS30	beclin 1
BEST1	BMD,BEST,RP50; vitelliform macular dystrophy 2; Best disease	bestrophin 1
BGN	DSPG1,SLRR1A; biglycan proteoglycan	biglycan
BID		BH3 interacting domain death agonist
BIRC5	EPR-1,survivin; apoptosis inhibitor 4,baculoviral IAP repeat-containing 5; survivin variant 3 alpha	baculoviral IAP repeat containing 5
BMP1	procollagen C-endopeptidase,tolloid-like; BMP-1; procollagen C-endopeptidase	bone morphogenetic protein 1
BMP2		bone morphogenetic protein 2
BMP3	bone morphogenetic protein 3 (osteogenic); osteogenin	bone morphogenetic protein 3
BMP4		bone morphogenetic protein 4
BMP5		bone morphogenetic protein 5
BMP6	vegetal related growth factor (TGFB-related); VGR1	bone morphogenetic protein 6
BMP7	osteogenic protein 1; OP-1	bone morphogenetic protein 7
BMPR1A	bone morphogenetic protein receptor, type IA; ALK3,CD292	bone morphogenetic protein receptor type 1A
BMPR2	primary pulmonary hypertension 1,bone morphogenetic protein receptor, type II (serine/threonine kinase),bone morphogenetic protein receptor type II; BRK-3,T-ALK,BMPR3,BMPR-II	bone morphogenetic protein receptor type 2
BMX	ETK,PSCTK3; BTK-like on X chromosome	BMX non-receptor tyrosine kinase
BRCA1	RNF53,BRCC1,PPP1R53,FANCS; BRCA1/BRCA2-containing complex, subunit 1,protein phosphatase 1, regulatory subunit 53,Fanconi anemia, complementation group S; breast cancer 1, early onset,breast cancer 1	BRCA1 DNA repair associated
BST1	NAD(+) nucleosidase,ADP-ribosyl cyclase 2; CD157,BST-1	bone marrow stromal cell antigen 1
BST2	antiviral factor tetherin; CD317,tetherin,BST-2,HM1.24	bone marrow stromal cell antigen 2
BTG1	B-cell translocation gene 1, anti-proliferative; APRO2	BTG anti-proliferation factor 1
BTK	Bruton's tyrosine kinase; Bruton agammaglobulinemia tyrosine kinase; ATK,XLA,PSCTK1	Bruton tyrosine kinase
C11orf96	AG2	chromosome 11 open reading frame 96
C1QA	complement component 1, q subcomponent, alpha polypeptide,complement component 1, q subcomponent, A chain,complement C1q chain A	complement C1q A chain
C1QB	complement component 1, q subcomponent, beta polypeptide,complement component 1, q subcomponent, B chain,complement C1q chain B	complement C1q B chain
C1QC	complement component 1, q subcomponent, gamma polypeptide,complement component 1, q subcomponent, C chain	complement C1q C chain
C5AR2	C5L2; G protein-coupled receptor 77	complement component 5a receptor 2
C9orf16	BBLN	bublin coiled coil protein
CALB1	calbindin 1, 28kDa	calbindin 1
CALD1	CDM,H-CAD,L-CAD	caldesmon 1
CALM1	calmodulin 1 (phosphorylase kinase, delta); CAMI,PHKD,DD132; prepro-calmodulin 1,phosphorylase kinase subunit delta	calmodulin 1
CALM2	prepro-calmodulin 2,phosphorylase kinase subunit delta; PHKD,CAMII; calmodulin 2 (phosphorylase kinase, delta)	calmodulin 2
CALM3	calmodulin 3 (phosphorylase kinase, delta); PHKD; prepro-calmodulin 3,phosphorylase kinase subunit delta	calmodulin 3

CAMP	CAP18,FALL39,FALL-39,LL37	cathelicidin antimicrobial peptide
CASP3	caspase 3, apoptosis-related cysteine protease,caspase 3, apoptosis-related cysteine peptidase; CPP32,CPP32B,Yama,apopain	caspase 3
CASP8	MCH5,MACH,FLICE,Casp-8; caspase 8, apoptosis-related cysteine protease,caspase 8, apoptosis-related cysteine peptidase	caspase 8
CASR	hypocalciuric hypercalcemia 1,calcium-sensing receptor; severe neonatal hyperparathyroidism; FHH,NSHPT,GPRC2A	calcium sensing receptor
CAV1	caveolin 1, caveolae protein, 22kD,caveolin 1, caveolae protein, 22kDa	caveolin 1
CCL11	small inducible cytokine subfamily A (Cys-Cys), member 11 (eotaxin),chemokine (C-C motif) ligand 11; eotaxin,MGC22554; eotaxin-1	C-C motif chemokine ligand 11
CCL13	small inducible cytokine subfamily A (Cys-Cys), member 13,chemokine (C-C motif) ligand 13; MCP-4,NCC-1,SCYL1,CKb10,MGC17134	C-C motif chemokine ligand 13
CCL15	leukotactin 1,CC chemokine 3,macrophage inflammatory protein 5,chemokine CC-2,MIP-1 delta; HCC-2,NCC-3,SCYL3,MIP-5,Lkn-1,MIP-1d,HMRP-2B; small inducible cytokine subfamily A (Cys-Cys), member 15,chemokine (C-C motif) ligand 15	C-C motif chemokine ligand 15
CCL18	DC-CK1,PARC,AMAC-1,DCCK1,MIP-4,CKb7; pulmonary and activation-regulated; small inducible cytokine subfamily A (Cys-Cys), member 18, pulmonary and activation-regulated,chemokine (C-C motif) ligand 18 (pulmonary and activation-regulated),chemokine (C-C motif) ligand 18	C-C motif chemokine ligand 18
CCL19	small inducible cytokine subfamily A (Cys-Cys), member 19,chemokine (C-C motif) ligand 19; CC chemokine ligand 19,macrophage inflammatory protein 3-beta,beta chemokine exodus-3,CK beta-11,EBI1-ligand chemokine; ELC,MIP-3b,exodus-3,CKb11	C-C motif chemokine ligand 19
CCL2	monocyte chemotactic protein 1, homologous to mouse Sig-je,monocyte chemoattractant protein-1,monocyte chemotactic and activating factor,monocyte secretory protein JE,small inducible cytokine subfamily A (Cys-Cys), member 2; small inducible cytokine A2 (monocyte chemotactic protein 1, homologous to mouse Sig-je),chemokine (C-C motif) ligand 2; MCP1,MCP-1,MCAF,SMC-CF,GDCF-2,HC11,MGC9434	C-C motif chemokine ligand 2
CCL20	small inducible cytokine subfamily A (Cys-Cys), member 20,chemokine (C-C motif) ligand 20; LARC,MIP-3a,exodus-1,ST38,CKb4	C-C motif chemokine ligand 20
CCL21	SLC,exodus-2,TCA4,CKb9,6Ckine,ECL; small inducible cytokine subfamily A (Cys-Cys), member 21,chemokine (C-C motif) ligand 21; beta chemokine exodus-2,secondary lymphoid tissue chemokine,Efficient Chemoattractant for Lymphocytes	C-C motif chemokine ligand 21
CCL23	small inducible cytokine subfamily A (Cys-Cys), member 23,chemokine (C-C motif) ligand 23; Ckb-8,MPIF-1,MIP-3,CKb8	C-C motif chemokine ligand 23
CCL26	MIP-4alpha,eotaxin-3,IMAC,MIP-4a,TSC-1; small inducible cytokine subfamily A (Cys-Cys), member 26,chemokine (C-C motif) ligand 26; macrophage inflammatory protein 4-alpha,small inducible cytokine A26,CC chemokine IMAC,chemokine N1,thymic stroma chemokine-1,eotaxin-3	C-C motif chemokine ligand 26
CCL28	SCYA28,MEC,CCK1; CC chemokine CCL28,mucosae-associated epithelial chemokine,small inducible cytokine subfamily A (Cys-Cys), member 28,small inducible cytokine A28; chemokine (C-C motif) ligand 28	C-C motif chemokine ligand 28
CCL3	small inducible cytokine A3 (homologous to mouse Mip-1a),chemokine (C-C motif) ligand 3; G0S19-1,LD78ALPHA,MIP-1-alpha	C-C motif chemokine ligand 3
CCL3L3	chemokine (C-C motif) ligand 3-like 3; MGC12815	C-C motif chemokine ligand 3 like 3

CCL4	MIP-1-beta,Act-2,AT744.1; small inducible cytokine A4 (homologous to mouse Mip-1b),chemokine (C-C motif) ligand 4	C-C motif chemokine ligand 4
CCL4L2	chemokine (C-C motif) ligand 4-like 2	C-C motif chemokine ligand 4 like 2
CCL5	RANTES,SISd,TCP228,MGC17164; T-cell specific protein p288,T-cell specific RANTES protein,SIS-delta,regulated upon activation, normally T-expressed, and presumably secreted,beta-chemokine RANTES,small inducible cytokine subfamily A (Cys-Cys), member 5; small inducible cytokine A5 (RANTES),chemokine (C-C motif) ligand 5	C-C motif chemokine ligand 5
CCL7	monocyte chemoattractant protein 3,monocyte chemotactic protein 3; MCP-3,NC28,FIC,MARC,MCP3; small inducible cytokine A7 (monocyte chemotactic protein 3),chemokine (C-C motif) ligand 7	C-C motif chemokine ligand 7
CCL8	MCP-2,HC14; small inducible cytokine subfamily A (Cys-Cys), member 8 (monocyte chemotactic protein 2),chemokine (C-C motif) ligand 8	C-C motif chemokine ligand 8
CCND1	cyclin D1 (PRAD1: parathyroid adenomatosis 1); U21B31; parathyroid adenomatosis 1,B-cell CLL/lymphoma 1,G1/S-specific cyclin D1	cyclin D1
CCR1	CKR-1,MIP1aR,CD191; chemokine (C-C motif) receptor 1	C-C motif chemokine receptor 1
CCR10	G protein-coupled receptor 2,chemokine (C-C motif) receptor 10	C-C motif chemokine receptor 10
CCR2	CC-CKR-2,CKR2,MCP-1-R,CD192,FLJ78302; chemokine (C-C motif) receptor 2	C-C motif chemokine receptor 2
CCR5	chemokine (C-C motif) receptor 5,chemokine (C-C motif) receptor 5 (gene/pseudogene),C-C motif chemokine receptor 5 (gene/pseudogene); CKR-5,CC-CKR-5,CKR5,CD195,IDDM22	C-C motif chemokine receptor 5
CCR7	chemokine (C-C motif) receptor 7; BLR2,CDw197,CD197	C-C motif chemokine receptor 7
CCRL2	HCR,CRAM-B,CKRX,CRAM-A,ACKR5; chemokine (C-C motif) receptor-like 2; atypical chemokine receptor 5	C-C motif chemokine receptor like 2
CD14	CD14 antigen	CD14 molecule
CD163	M130,MM130,SCARI1; CD163 antigen	CD163 molecule
CD164	deafness, autosomal dominant 66; MUC-24,MGC-24,DFNA66; CD164 antigen, sialomucin,CD164 molecule, sialomucin	CD164 molecule
CD19	CD19 antigen	CD19 molecule
CD2	CD2 antigen (p50), sheep red blood cell receptor	CD2 molecule
CD209	CD209 antigen; DC-SIGN,CDSIGN,DC-SIGN1,CLEC4L	CD209 molecule
CD24	CD24 antigen (small cell lung carcinoma cluster 4 antigen); CD24A	CD24 molecule
CD27	S152,Tp55; tumor necrosis factor receptor superfamily, member 7	CD27 molecule
CD274	B7-H,B7H1,PD-L1,PDL1,B7-H1; programmed cell death 1 ligand 1,CD274 antigen; B7 homolog 1	CD274 molecule
CD276	CD276 antigen; B7-H3,B7H3,B7RP-2	CD276 molecule
CD28	CD28 antigen (Tp44); T-cell-specific surface glycoprotein	CD28 molecule
CD300A	CD300a antigen; Irp60,CMRF35H,CMRF-35-H9,IRC1,IRC2,IGSF12	CD300a molecule
CD33	sialic acid binding Ig-like lectin 3; CD33 antigen (gp67); SIGLEC3,SIGLEC-3,p67,FLJ00391	CD33 molecule
CD34	CD34 antigen	CD34 molecule
CD36	CD36 antigen (collagen type I receptor, thrombospondin receptor),CD36 molecule (thrombospondin receptor); SCARB3,GPIV,FAT,GP4,GP3B	CD36 molecule
CD37	CD37 antigen; TSPAN26	CD37 molecule
CD38	ADP-ribosyl cyclase 1,NAD(+) nucleosidase; CD38 antigen (p45)	CD38 molecule
CD3D	CD3d antigen, delta polypeptide (TiT3 complex),CD3d molecule, delta (CD3-TCR complex)	CD3d molecule
CD3E	CD3e antigen, epsilon polypeptide (TiT3 complex),CD3e molecule, epsilon (CD3-TCR complex)	CD3e molecule
CD3G	CD3g antigen, gamma polypeptide (TiT3 complex),CD3g molecule, gamma (CD3-TCR complex)	CD3g molecule

CD4	CD4 antigen (p55),T-cell surface glycoprotein CD4	CD4 molecule
CD40	tumor necrosis factor receptor superfamily, member 5,CD40 molecule, TNF receptor superfamily member 5; p50,Bp50	CD40 molecule
CD40LG	CD40L,TRAP,gp39,hCD40L,CD154; tumor necrosis factor (ligand) superfamily, member 5 (hyper-IgM syndrome); CD40 antigen ligand,tumor necrosis factor (ligand) superfamily member 5,T-B cell-activating molecule,TNF-related activation protein,hyper-IgM syndrome	CD40 ligand
CD44	CD44 antigen (homing function and Indian blood group system); IN,MC56,Pgp1,CD44R,HCELL,CSPG8; hematopoietic cell E- and L-selectin ligand,chondroitin sulfate proteoglycan 8	CD44 molecule (Indian blood group)
CD47	antigen identified by monoclonal antibody 1D8,antigenic surface determinant protein OA3,integrin associated protein,Rh-related antigen,leukocyte surface antigen CD47,CD47 glycoprotein; IAP,OA3; CD47 antigen (Rh-related antigen, integrin-associated signal transducer)	CD47 molecule
CD48	BLAST,mCD48,hCD48,SLAMF2; CD48 antigen (B-cell membrane protein),CD48 molecule	CD48 molecule
CD52	HE5,EDDM5; CD52 antigen (CAMPATH-1 antigen)	CD52 molecule
CD53	CD53 antigen; TSPAN25	CD53 molecule
CD55	CR,TC,CROM; decay accelerating factor for complement (CD55, Cromer blood group system),CD55 molecule, decay accelerating factor for complement (Cromer blood group)	CD55 molecule (Cromer blood group)
CD58	CD58 antigen, (lymphocyte function-associated antigen 3)	CD58 molecule
CD59	16.3A5,EJ16,EJ30,EL32,G344,p18-20; CD59 antigen p18-20 (antigen identified by monoclonal antibodies 16.3A5, EJ16, EJ30, EL32 and G344),CD59 antigen, complement regulatory protein,CD59 molecule, complement regulatory protein,CD59 molecule	CD59 molecule (CD59 blood group)
CD5L	apoptosis inhibitor 6,CD5 antigen-like (scavenger receptor cysteine rich family),CD5 molecule-like; Spalpha	CD5 molecule like
CD63	ME491,TSPAN30; CD63 antigen (melanoma 1 antigen)	CD63 molecule
CD68	scavenger receptor class D, member 1,CD68 antigen,macrophage antigen CD68; SCARD1,macrosialin,GP110,DKFZp686M18236,LAMP4; CD68 antigen	CD68 molecule
CD69	CLEC2C; CD69 antigen (p60, early T-cell activation antigen)	CD69 molecule
CD70	tumor necrosis factor (ligand) superfamily, member 7; CD27L	CD70 molecule
CD74	CD74 antigen (invariant polypeptide of major histocompatibility complex, class II antigen-associated),CD74 molecule, major histocompatibility complex, class II invariant chain; HLA-DR-gamma,Ia-associated invariant chain,gamma chain of class II antigens,MHC HLA-DR gamma chain	CD74 molecule
CD79A	CD79A antigen (immunoglobulin-associated alpha),CD79a molecule, immunoglobulin-associated alpha; B-cell antigen receptor complex-associated protein alpha chain; MB-1	CD79a molecule
CD80	B-lymphocyte activation antigen B7; B7.1,B7-1; CD80 antigen (CD28 antigen ligand 1, B7-1 antigen),CD80 molecule	CD80 molecule
CD81	CD81 antigen (target of antiproliferative antibody 1); TAPA-1,TSPAN28	CD81 molecule
CD83	HB15,BL11; CD83 antigen (activated B lymphocytes, immunoglobulin superfamily),CD83 molecule	CD83 molecule
CD84	CD84 antigen (leukocyte antigen),CD84 molecule ; SLAMF5,hCD84,mCD84	CD84 molecule
CD86	B-lymphocyte antigen B7-2; B7.2,B7-2; CD86 antigen (CD28 antigen ligand 2, B7-2 antigen)	CD86 molecule
CD8A	CD8 antigen, alpha polypeptide (p32),T-cell surface glycoprotein CD8 alpha chain	CD8a molecule
CD8B	CD8 antigen, beta polypeptide 1 (p37)	CD8b molecule

CD9	CD9 antigen (p24); motility related protein-1; BA2,P24,TSPAN29,MRP-1	CD9 molecule
CDH1	E-Cadherin; cadherin 1, type 1, E-cadherin (epithelial); uvomorulin,CD324	cadherin 1
CDH11	OB,CAD11; OB-Cadherin; cadherin 11, type 2, OB-cadherin (osteoblast)	cadherin 11
CDH5	cadherin 5, type 2, VE-cadherin (vascular epithelium),cadherin 5, type 2 (vascular endothelium); VE-cadherin; 7B4,CD144	cadherin 5
CDKN1A	P21,CIP1,WAF1,SDI1,CAP20,p21CIP1,p21Cip1/Waf1,p21; cyclin-dependent kinase inhibitor 1A (p21, Cip1)	cyclin dependent kinase inhibitor 1A
CDKN3	kinase associated phosphatase,cyclin-dependent kinase inhibitor,CDK2-associated dual specificity phosphatase; KAP,CDI1	cyclin dependent kinase inhibitor 3
CEACAM1	BGP1,CD66a; carcinoembryonic antigen-related cell adhesion molecule 1 (biliary glycoprotein),carcinoembryonic antigen related cell adhesion molecule 1	CEA cell adhesion molecule 1
CEACAM6	CD66c; carcinoembryonic antigen-related cell adhesion molecule 6 (non-specific cross reacting antigen),carcinoembryonic antigen related cell adhesion molecule 6	CEA cell adhesion molecule 6
CELSR1	cadherin, EGF LAG seven-pass G-type receptor 1, flamingo (Drosophila) homolog,cadherin, EGF LAG seven-pass G-type receptor 1; ME2,HFMI2,FMI2,CDHF9,ADGRC1; flamingo homolog 2 (Drosophila),adhesion G protein-coupled receptor C1	cadherin EGF LAG seven-pass G-type receptor 1
CELSR2	adhesion G protein-coupled receptor C2; KIAA0279,MEGF3,Flamingo1,CDHF10,ADGRC2; cadherin, EGF LAG seven-pass G-type receptor 2, flamingo (Drosophila) homolog,cadherin, EGF LAG seven-pass G-type receptor 2	cadherin EGF LAG seven-pass G-type receptor 2
CENPF	hcp-1; centromere protein F, 350/400kDa (mitosin),centromere protein F, 350/400kDa; mitosin	centromere protein F
CFD	D component of complement (adipsin),properdin factor D,complement factor D (adipsin); adipsin; ADN	complement factor D
CFLAR	CASH,Casper,CLARP,FLAME,FLIP,I-FLICE,MRIT,c-FLIP,cFLIP; CASP8 and FADD-like apoptosis regulator	CASP8 and FADD like apoptosis regulator
CHEK1	CHK1; CHK1 (checkpoint, S.pombe) homolog,CHK1 checkpoint homolog (S. pombe)	checkpoint kinase 1
CHEK2	CHK2 (checkpoint, S.pombe) homolog,CHK2 checkpoint homolog (S. pombe); CDS1,CHK2,HuCds1,PP1425,bA444G7	checkpoint kinase 2
CHGA	vasostatin,pancreastatin,parastatin,parathyroid secretory protein 1	chromogranin A
CHI3L1	chitinase 3-like 1 (cartilage glycoprotein-39); cartilage glycoprotein-39; GP39,YKL40,YK-40	chitinase 3 like 1
CIDEA	cell death-inducing DFFA-like effector a; CIDE-A	cell death inducing DFFA like effector a
CIITA	MHC class II transactivator,class II, major histocompatibility complex, transactivator; NLR family, acid domain containing,nucleotide-binding oligomerization domain, leucine rich repeat and acid domain containing; C2TA,NLRA	class II major histocompatibility complex transactivator
CLCF1	B-cell stimulating factor 3,cold-induced sweating syndrome 2,novel neurotrophin-1; CRLF1 associated cytokine-like factor 1; NNT1,BSF3,CLC,NR6,CISS2,BSF-3,NNT-1	cardiotrophin like cytokine factor 1
CLDN4	Clostridium perfringens enterotoxin receptor 1,Williams-Beuren syndrome chromosomal region 8 protein; CPE-R,WBSCR8,hCPE-R	claudin 4
CLEC10A	HML2,HML,CD301; C-type (calcium dependent, carbohydrate-recognition domain) lectin, superfamily member 14 (macrophage-derived),C-type lectin domain family 10, member A,C-type lectin domain family 10 member A; macrophage lectin 2 (calcium dependent)	C-type lectin domain containing 10A
CLEC12A	C-type lectin domain family 12, member A; dendritic cell-associated lectin 2,myeloid inhibitory C-type lectin-like receptor; CLL-1,MICL,CD371,DCAL-2	C-type lectin domain family 12 member A

CLEC14A	chromosome 14 open reading frame 27,C-type lectin domain family 14, member A,C-type lectin domain family 14 member A	C-type lectin domain containing 14A
CLEC1A	C-type lectin domain family 1, member A; CLEC1,MGC34328	C-type lectin domain family 1 member A
CLEC2B	C-type (calcium dependent, carbohydrate-recognition domain) lectin, superfamily member 2 (activation-induced),C-type lectin domain family 2, member B; AICL,HP10085	C-type lectin domain family 2 member B
CLEC2D	C-type lectin related f,lectin-like transcript 1; LLT1,CLAX,OCIL; C-type lectin superfamily 2, member D,C-type lectin domain family 2, member D	C-type lectin domain family 2 member D
CLEC4A	DCIR,DDB27,CD367; C-type (calcium dependent, carbohydrate-recognition domain) lectin, superfamily member 6,C-type lectin domain family 4, member A	C-type lectin domain family 4 member A
CLEC4D	Dectin 3; C-type (calcium dependent, carbohydrate-recognition domain) lectin, superfamily member 8,C-type lectin domain family 4, member D; Mpcl,CD368,MCL,Dectin-3	C-type lectin domain family 4 member D
CLEC4E	C-type (calcium dependent, carbohydrate-recognition domain) lectin, superfamily member 9,C-type lectin domain family 4, member E; Macrophage-inducible C-type lectin; mincle	C-type lectin domain family 4 member E
CLEC5A	C-type (calcium dependent, carbohydrate-recognition domain) lectin, superfamily member 5,C-type lectin domain family 5, member A,C-type lectin domain family 5 member A; MDL-1	C-type lectin domain containing 5A
CLEC7A	dectin-1,hDectin-1,CD369,SCARE2; C-type (calcium dependent, carbohydrate-recognition domain) lectin, superfamily member 12,C-type lectin domain family 7, member A,C-type lectin domain family 7 member A	C-type lectin domain containing 7A
CLOCK	clock (mouse) homolog,clock homolog (mouse); KIAA0334,KAT13D,bHLHe8	clock circadian regulator
CLU	SGP-2,SP-40,TRPM-2,KUB1,CLU1,CLU2; complement lysis inhibitor,sulfated glycoprotein 2,testosterone-repressed prostate message 2,apolipoprotein J; clusterin (complement lysis inhibitor, SP-40,40, sulfated glycoprotein 2, testosterone-repressed prostate message 2, apolipoprotein J)	clusterin
CMKLR1	chemokine-like receptor 1; RVER1; resolvin E1 receptor,chemerin receptor	chemerin chemokine-like receptor 1
CNTFR		ciliary neurotrophic factor receptor
COL11A1	collagen, type XI, alpha 1,deafness, autosomal dominant 37; collagen XI, alpha-1 polypeptide; STL2,CO11A1	collagen type XI alpha 1 chain
COL12A1	collagen type XII proteoglycan; collagen, type XII, alpha 1-like,collagen, type XII, alpha 1	collagen type XII alpha 1 chain
COL14A1	undulin,collagen, type XIV, alpha 1	collagen type XIV alpha 1 chain
COL15A1	collagen type XV proteoglycan; collagen, type XV, alpha 1	collagen type XV alpha 1 chain
COL16A1	collagen, type XVI, alpha 1	collagen type XVI alpha 1 chain
COL17A1	BP180; collagen, type XVII, alpha 1	collagen type XVII alpha 1 chain
COL18A1	Knobloch syndrome, type 1,collagen, type XVIII, alpha 1; endostatin; KS,KNO1	collagen type XVIII alpha 1 chain
COL1A1	OI4; collagen, type I, alpha 1	collagen type I alpha 1 chain
COL1A2	alpha 2(I)-collagen,alpha-2 collagen type I,type I procollagen,collagen I, alpha-2 polypeptide,collagen of skin, tendon and bone, alpha-2 chain; osteogenesis imperfecta type IV,collagen, type I, alpha 2,collagen type I alpha 2	collagen type I alpha 2 chain
COL21A1	collagen, type XXI, alpha 1	collagen type XXI alpha 1 chain
COL27A1	KIAA1870,MGC11337,FLJ11895; collagen, type XXVII, alpha 1,collagen type XXVII alpha 1	collagen type XXVII alpha 1 chain
COL3A1	Ehlers-Danlos syndrome type IV, autosomal dominant,collagen, type III, alpha 1	collagen type III alpha 1 chain
COL4A1	collagen, type IV, alpha 1	collagen type IV alpha 1 chain
COL4A2	canstatin,collagen type IV alpha 2; FLJ22259,DKFZp686I14213; collagen type IV alpha 2	collagen type IV alpha 2 chain

COL4A5	Alport syndrome,collagen, type IV, alpha 5,collagen type IV alpha 5	collagen type IV alpha 5 chain
COL5A1	collagen type V alpha 1; alpha 1 type V collagen	collagen type V alpha 1 chain
COL5A2	collagen, type V, alpha 2; AB collagen	collagen type V alpha 2 chain
COL5A3	collagen type V alpha 3	collagen type V alpha 3 chain
COL6A1	collagen, type VI, alpha 1	collagen type VI alpha 1 chain
COL6A2	collagen type VI alpha 2	collagen type VI alpha 2 chain
COL6A3	collagen, type VI, alpha 3	collagen type VI alpha 3 chain
COL8A1	MGC9568; chromosome 3 open reading frame 7,collagen, type VIII, alpha 1	collagen type VIII alpha 1 chain
COL9A1	collagen, type IX, alpha 1	collagen type IX alpha 1 chain
COL9A2	MED; collagen, type IX, alpha 2	collagen type IX alpha 2 chain
COL9A3	collagen, type IX, alpha 3; IDD,MED,EDM3,FLJ90759,DJ885L7.4.1; collagen type IX proteoglycan	collagen type IX alpha 3 chain
COTL1	coactosin-like 1 (Dictyostelium),coactosin-like F-actin binding protein 1; CLP	coactosin like F-actin binding protein 1
CPA3	carboxypeptidase A3 (mast cell); mast cell carboxypeptidase A,tissue carboxypeptidase A	carboxypeptidase A3
CPB1	pancreatic carboxypeptidase B,tissue carboxypeptidase B,protaminase; carboxypeptidase B1 (tissue)	carboxypeptidase B1
CRIP1	CRIP; cysteine-rich protein 1 (intestinal)	cysteine rich protein 1
CRP	C-reactive protein, pentraxin-related; pentraxin 1; PTX1	C-reactive protein
CRYAB	HSPB5; crystallin, alpha B	crystallin alpha B
CSF1	M-CSF,MCSF,MGC31930; colony stimulating factor 1 (macrophage); macrophage colony stimulating factor 1	colony stimulating factor 1
CSF1R	C-FMS,CSFR,CD115; McDonough feline sarcoma viral (v-fms) oncogene homolog	colony stimulating factor 1 receptor
CSF2	GM-CSF,GMCSF; sargramostim,molgramostim,granulocyte-macrophage colony stimulating factor; colony stimulating factor 2 (granulocyte-macrophage)	colony stimulating factor 2
CSF2RA	alpha-GM-CSF receptor; colony stimulating factor 2 receptor, alpha, low-affinity (granulocyte-macrophage); CD116,alphaGMR	colony stimulating factor 2 receptor subunit alpha
CSF2RB	beta common cytokine receptor,beta-GM-CSF receptor; IL5RB,CD131,betaGMR; colony stimulating factor 2 receptor, beta, low-affinity (granulocyte-macrophage),colony stimulating factor 2 receptor beta common subunit	colony stimulating factor 2 receptor subunit beta
CSF3	granulocyte colony stimulating factor,pluripoietin,filgrastim,lenograstim; chromosome 17 open reading frame 33,colony stimulating factor 3 (granulocyte); MGC45931	colony stimulating factor 3
CSF3R	colony stimulating factor 3 receptor (granulocyte); GCSFR	colony stimulating factor 3 receptor
CSHL1	chorionic somatomammotropin CS-5; hCS-L,CSL,CS-5,MGC149868	chorionic somatomammotropin hormone like 1
CSK	c-src tyrosine kinase,CSK, non-receptor tyrosine kinase	C-terminal Src kinase
CST7	cystatin F (leukocystatin); leukocystatin	cystatin F
CTLA4	CD152,CD,GSE,CTLA-4; celiac disease 3,insulin-dependent diabetes mellitus 12	cytotoxic T-lymphocyte associated protein 4
CTNNB1	catenin (cadherin-associated protein), beta 1 (88kD),catenin (cadherin-associated protein), beta 1, 88kDa,catenin (cadherin-associated protein), beta 1; beta-catenin,armadillo	catenin beta 1
CTSG	CG	cathepsin G
CTSW	cathepsin W (lymphopain)	cathepsin W
CUZD1	ERG-1,UO-44; CUB and zona pellucida-like domains 1	CUB and zona pellucida like domains 1
CX3CL1	NTN,C3Xkine,ABCD-3,CXC3C,CXC3; small inducible cytokine subfamily D (Cys-X3-Cys), member 1 (fractalkine, neurotactin),chemokine (C-X3-C motif) ligand 1; fractalkine,neurotactin	C-X3-C motif chemokine ligand 1
CX3CR1	CMKDR1,V28,CCRL1; chemokine (C-X3-C) receptor 1,chemokine (C-X3-C motif) receptor 1	C-X3-C motif chemokine receptor 1

CXCL1	SCYB1,GROa,MGSA-a,NAP-3; GRO1 oncogene (melanoma growth stimulating activity, alpha),fibroblast secretory protein,chemokine (C-X-C motif) ligand 1 (melanoma growth stimulating activity, alpha); melanoma growth stimulating activity, alpha	C-X-C motif chemokine ligand 1
CXCL10	IFI10,IP-10,crg-2,mob-1,C7,gIP-10; small inducible cytokine subfamily B (Cys-X-Cys), member 10,chemokine (C-X-C motif) ligand 10	C-X-C motif chemokine ligand 10
CXCL12	SCYB12,SDF-1a,SDF-1b,PBSF,TLSF-a,TLSF-b,TPAR1; stromal cell-derived factor 1,chemokine (C-X-C motif) ligand 12	C-X-C motif chemokine ligand 12
CXCL14	BRAK,NJAC,bolekine,Kec,MIP-2g,BMAC,KS1; small inducible cytokine subfamily B (Cys-X-Cys), member 14 (BRAK),chemokine (C-X-C motif) ligand 14; breast and kidney	C-X-C motif chemokine ligand 14
CXCL16	chemokine (C-X-C motif) ligand 16; CXC chemokine ligand 16; SR-PSOX,CXCLG16,SRPSOX	C-X-C motif chemokine ligand 16
CXCL17	Dcip1,UNQ473,DMC,VCC1; chemokine (C-X-C motif) ligand 17	C-X-C motif chemokine ligand 17
CXCL2	SCYB2,GROb,MIP-2a,MGSA-b,CINC-2a; GRO2 oncogene,chemokine (C-X-C motif) ligand 2	C-X-C motif chemokine ligand 2
CXCL3	SCYB3,GROg,MIP-2b,CINC-2b; GRO3 oncogene,chemokine (C-X-C motif) ligand 3	C-X-C motif chemokine ligand 3
CXCL5	ENA-78; small inducible cytokine subfamily B (Cys-X-Cys), member 5 (epithelial-derived neutrophil-activating peptide 78),chemokine (C-X-C motif) ligand 5	C-X-C motif chemokine ligand 5
CXCL6	GCP-2,CKA-3; granulocyte chemotactic protein 2; small inducible cytokine subfamily B (Cys-X-Cys), member 6 (granulocyte chemotactic protein 2),chemokine (C-X-C motif) ligand 6 (granulocyte chemotactic protein 2),chemokine (C-X-C motif) ligand 6	C-X-C motif chemokine ligand 6
CXCL8	interleukin 8,chemokine (C-X-C motif) ligand 8; neutrophil-activating peptide 1,granulocyte chemotactic protein 1,monocyte-derived neutrophil chemotactic factor,lung giant cell carcinoma-derived chemotactic protein,tumor necrosis factor-induced gene 1,monocyte-derived neutrophil-activating peptide,lymphocyte derived neutrophil activating peptide,beta endothelial cell-derived neutrophil activating peptide,alveolar macrophage chemotactic factor I; SCYB8,LUCT,LECT,MDNCF,TSG-1,IL-8,NAP-1,3-10C,MONAP,AMCF-I,LYNAP,NAF,b-ENAP,GCP-1,K60,GCP1,NAP1	C-X-C motif chemokine ligand 8
CXCL9	monokine induced by gamma interferon,chemokine (C-X-C motif) ligand 9; SCYB9,Humig,crg-10	C-X-C motif chemokine ligand 9
CXCR1	CKR-1,CDw128a,CD181; interleukin 8 receptor, alpha,chemokine (C-X-C motif) receptor 1	C-X-C motif chemokine receptor 1
CXCR2	CMKAR2,CD182; interleukin 8 receptor, beta,chemokine (C-X-C motif) receptor 2	C-X-C motif chemokine receptor 2
CXCR3	G protein-coupled receptor 9,chemokine (C-X-C motif) receptor 3; CKR-L2,CMKAR3,IP10-R,MigR,CD183	C-X-C motif chemokine receptor 3
CXCR4	LESTR,NPY3R,HM89,NPYY3R,D2S201E,fusin,HSY3RR,NPYR,CD184; chemokine (C-X-C motif), receptor 4 (fusin),chemokine (C-X-C motif) receptor 4	C-X-C motif chemokine receptor 4
CXCR5	Burkitt lymphoma receptor 1, GTP-binding protein,Burkitt lymphoma receptor 1, GTP binding protein (chemokine (C-X-C motif) receptor 5),chemokine (C-X-C motif) receptor 5; MDR15,CD185	C-X-C motif chemokine receptor 5
CXCR6	chemokine (C-X-C motif) receptor 6; TYMSTR,STRL33,BONZO,CD186	C-X-C motif chemokine receptor 6
CYP19A1	cytochrome P450, subfamily XIX (aromatization of androgens),cytochrome P450, family 19, subfamily A, polypeptide 1; ARO,P-450AROM,CPV1,ARO1,CYAR,aromatase	cytochrome P450 family 19 subfamily A member 1
CYP1B1	cytochrome P450, subfamily I (dioxin-inducible), polypeptide 1 (glaucoma 3, primary infantile),cytochrome P450, family 1, subfamily B, polypeptide 1; CP1B	cytochrome P450 family 1 subfamily B member 1

CYSTM1	ORF1-FL49; chromosome 5 open reading frame 32	cysteine rich transmembrane module containing 1
CYTOR	MGC4677; chromosome 2 open reading frame 59,non-protein coding RNA 152,long intergenic non-protein coding RNA 152	cytoskeleton regulator RNA
DCN	decorin proteoglycan; DSPG2,SLRR1B	decorin
DDC	AADC; aromatic L-amino acid decarboxylase	dopa decarboxylase
DDIT3	CHOP10,GADD153,CHOP; C/EBP zeta; DNA-damage-inducible transcript 3	DNA damage inducible transcript 3
DDR1	RTK6,CD167; discoidin domain receptor family, member 1	discoidin domain receptor tyrosine kinase 1
DDR2	TKT; discoidin domain receptor family, member 2	discoidin domain receptor tyrosine kinase 2
DDX58	RNA helicase RIG-I,retinoic acid inducible gene I; DEAD (Asp-Glu-Ala-Asp) box polypeptide 58; RIG-I,FLJ13599,DKFZp434J1111,RIG-1,RIG1	DExD/H-box helicase 58
DHRS2	HEP27,SDR25C1; short chain dehydrogenase/reductase family 25C, member 1; dehydrogenase/reductase (SDR family) member 2	dehydrogenase/reductase 2
DLL1	delta (Drosophila)-like 1,delta-like 1 (Drosophila)	delta like canonical Notch ligand 1
DMBT1	GP340,muclin,SALSA,Gp-340,hensin,vomeroglandin; salivary agglutinin,salivary scavenger and agglutinin	deleted in malignant brain tumors 1
DNMT1	DNA (cytosine-5-)-methyltransferase 1; MCMT,CXXC9	DNA methyltransferase 1
DNMT3A	DNA (cytosine-5-)-methyltransferase 3 alpha	DNA methyltransferase 3 alpha
DNTT	TDT; deoxynucleotidyltransferase, terminal; Terminal deoxynucleotidyltransferase	DNA nucleotidylexotransferase
DPP4	dipeptidylpeptidase IV (CD26, adenosine deaminase complexing protein 2),adenosine deaminase complexing protein 2,dipeptidyl-peptidase 4; DPPIV	dipeptidyl peptidase 4
DST	BP240,KIAA0728,FLJ21489,FLJ13425,FLJ32235,FLJ30627,CATX-15,BPA,MACF2; bullous pemphigoid antigen 1, 230/240kDa	dystonin
DUSP1	HVH1,CL100,MKP-1	dual specificity phosphatase 1
DUSP2	PAC-1	dual specificity phosphatase 2
DUSP4	HVH2,MKP-2,TYP; VH1 homologous phosphatase 2,MAP kinase phosphatase 2	dual specificity phosphatase 4
DUSP5	HVH3	dual specificity phosphatase 5
DUSP6	MKP-3,PYST1	dual specificity phosphatase 6
EFNA1	EPH-related receptor tyrosine kinase ligand 1,Tumor necrosis factor alpha-induced protein 4,gastric cancer metastasis associated long noncoding RNA; LERK1,ECKLG,GMAN; ephrin-A1	ephrin A1
EFNA4	ephrin-A4; LERK4; EPH-related receptor tyrosine kinase ligand 4	ephrin A4
EFNA5	EPH-related receptor tyrosine kinase ligand 7; ephrin-A5; AF1,LERK7	ephrin A5
EFNB1	LERK2,Elk-L; EPH-related receptor tyrosine kinase ligand 2; craniofrontonasal syndrome (craniofrontonasal dysplasia),ephrin-B1	ephrin B1
EFNB2	ephrin-B2; HTK ligand,ligand of eph-related kinase 5,eph-related receptor tyrosine kinase ligand 5; LERK5,Htk-L,HTKL,MGC126226,MGC126227,MGC126228	ephrin B2
EFNB3	ephrin-B3; LERK-8; EPH-related receptor tyrosine kinase ligand 8	ephrin B3
EGF	Pro-epidermal growth factor; epidermal growth factor (beta-urogastrone)	epidermal growth factor
EGFR	erythroblastic leukemia viral (v-erb-b) oncogene homolog (avian),erb-b2 receptor tyrosine kinase 1; epidermal growth factor receptor (avian erythroblastic leukemia viral (v-erb-b) oncogene homolog); ERBB1,ERRP	epidermal growth factor receptor
EIF5A	EIF5A1,EIF-5A,MGC99547,MGC104255	eukaryotic translation initiation factor 5A
ELANE	neutrophil elastase,leukocyte elastase,medullasin,PMN Elastase,polymorphonuclear leukocyte elastase; elastase 2, neutrophil; NE,HNE,HLE,PMN-E	elastase, neutrophil expressed
EMP3	YMP	epithelial membrane protein 3
ENG	Osler-Rendu-Weber syndrome 1; END,HHT1,CD105	endoglin
ENTPD1	NTPDase-1,ATPDase,SPG64	ectonucleoside triphosphate diphosphohydrolase 1
EOMES	T-box brain2; TBR2; eomesodermin (Xenopus laevis) homolog	eomesodermin

EPCAM	antigen identified by monoclonal antibody AUA1,tumor-associated calcium signal transducer 1; trophoblast cell surface antigen 1; Ly74,TROP1,GA733-2,EGP34,EGP40,EGP-2,KSA,CD326,Ep-CAM,HEA125,KS1/4,MK-1,MH99,MOC31,323/A3,17-1A,TACST-1,CO-17A,ESA	epithelial cell adhesion molecule
EPHA2	EphA2	EPH receptor A2
EPHA3	EphA3; HEK,HEK4	EPH receptor A3
EPHA4	Hek8; EphA4	EPH receptor A4
EPHA7	Hek11; EphA7	EPH receptor A7
EPHB2	Hek5,Tyro5; EphB2	EPH receptor B2
EPHB3	EphB3; Hek2,Tyro6	EPH receptor B3
EPHB4	EphB4; Tyro11	EPH receptor B4
EPHB6	HEP; EphB6	EPH receptor B6
EPOR		erythropoietin receptor
ERBB2	NEU,HER-2,CD340,HER2; neuro/glioblastoma derived oncogene homolog,human epidermal growth factor receptor 2; v-erb-b2 avian erythroblastic leukemia viral oncogene homolog 2 (neuro/glioblastoma derived oncogene homolog),v-erb-b2 avian erythroblastic leukemia viral oncogene homolog 2	erb-b2 receptor tyrosine kinase 2
ERBB3	lethal congenital contracture syndrome 2,v-erb-b2 avian erythroblastic leukemia viral oncogene homolog 3; human epidermal growth factor receptor 3; HER3	erb-b2 receptor tyrosine kinase 3
ESAM	W117m	endothelial cell adhesion molecule
ESR1	NR3A1,Era,ER-alpha; nuclear receptor subfamily 3 group A member 1,estrogen receptor alpha,oestrogen receptor alpha,E2 receptor alpha	estrogen receptor 1
ETS1	FLJ10768,ETS-1; v-ets avian erythroblastosis virus E26 oncogene homolog 1; Avian erythroblastosis virus E26 (v-ets) oncogene homolog-1,ets protein	ETS proto-oncogene 1, transcription factor
ETV4	E1A-F,E1AF,PEA3; E1A enhancer binding protein; ets variant gene 4 (E1A enhancer-binding protein, E1AF),ETS variant 4	ETS variant transcription factor 4
ETV5	ets-related molecule; ets variant gene 5 (ets-related molecule),ETS variant 5; ERM	ETS variant transcription factor 5
EZH2	EZH1,ENX-1,KMT6,KMT6A; enhancer of zeste (Drosophila) homolog 2,enhancer of zeste homolog 2 (Drosophila)	enhancer of zeste 2 polycomb repressive complex 2 subunit
EZR	cytovillin 2; villin 2 (ezrin)	ezrin
FABP4	A-FABP,aP2; adipocyte fatty acid binding protein	fatty acid binding protein 4
FABP5	E-FABP,PA-FABP,KFABP; fatty acid binding protein 5 (psoriasis-associated)	fatty acid binding protein 5
FAS	CD95,APO-1; TNF receptor superfamily member 6; tumor necrosis factor receptor superfamily, member 6,Fas (TNF receptor superfamily, member 6)	Fas cell surface death receptor
FASLG	tumor necrosis factor (ligand) superfamily, member 6,Fas ligand (TNF superfamily, member 6); FasL,CD178	Fas ligand
FASN	short chain dehydrogenase/reductase family 27X, member 1; FAS,SDR27X1	fatty acid synthase
FCER1G	Fc epsilon receptor Ig; Fc fragment of IgE, high affinity I, receptor for; gamma polypeptide	Fc fragment of IgE receptor Ig
FCGBP	IgG Fc binding protein,Human Fc gamma BP; FC(GAMMA)BP	Fc fragment of IgG binding protein
FCGR3A	Fc gamma receptor IIIa; Fc fragment of IgG, low affinity IIIa, receptor for (CD16),Fc fragment of IgG, low affinity IIIa, receptor (CD16a); CD16,CD16a	Fc fragment of IgG receptor IIIa
FCRLA	MGC4595,FCRLc2,FCRLb,FCRLc1,FCRLd,FCRLe,FCRL,FREB,FCRLX; Fc receptor-like and mucin-like 1,Fc receptor-like A	Fc receptor like A
FES	Oncogene FES, feline sarcoma virus,c-fes/fps protein; FPS; feline sarcoma (Snyder-Theilen) viral (v-fes)/Fujinami avian sarcoma (PRCII) viral (v-fps) oncogene homolog,feline sarcoma oncogene	FES proto-oncogene, tyrosine kinase
FFAR2	G protein-coupled receptor 43; FFA2R	free fatty acid receptor 2
FFAR3	G protein-coupled receptor 41; FFA3R	free fatty acid receptor 3
FFAR4	G protein-coupled receptor 129,G protein-coupled receptor 120,omega-3 fatty acid receptor 1; PGR4	free fatty acid receptor 4

FGF1	heparin-binding growth factor 1,endothelial cell growth factor, alpha,endothelial cell growth factor, beta; AFGF,ECGF,ECGFA,ECGFB,HBGF1,ECGF-beta,FGF-alpha,GLIO703; fibroblast growth factor 1 (acidic)	fibroblast growth factor 1
FGF12	fibroblast growth factor 12B,fibroblast growth factor homologous factor 1,myocyte-activating factor,fibroblast growth factor FGF-12b; FHF1	fibroblast growth factor 12
FGF13	fibroblast growth factor homologous factor 2; long intergenic non-protein coding RNA 889; FHF2,FGF2,FLJ30672	fibroblast growth factor 13
FGF18	FGF-18,ZFGF5	fibroblast growth factor 18
FGF2	fibroblast growth factor 2 (basic)	fibroblast growth factor 2
FGF7	fibroblast growth factor 7 (keratinocyte growth factor); KGF; keratinocyte growth factor	fibroblast growth factor 7
FGF9	fibroblast growth factor 9 (glia-activating factor); glia-activating factor	fibroblast growth factor 9
FGFR1	H2,H3,H4,H5,CEK,FLG,BFGFR,N-SAM,CD331; fms-related tyrosine kinase 2; Pfeiffer syndrome	fibroblast growth factor receptor 1
FGFR2	Crouzon syndrome,Pfeiffer syndrome; CEK3,TK14,TK25,ECT1,K-SAM,CD332; bacteria-expressed kinase,keratinocyte growth factor receptor,craniofacial dysostosis 1,Jackson-Weiss syndrome	fibroblast growth factor receptor 2
FGFR3	CEK2,JTK4,CD333; achondroplasia, thanatophoric dwarfism	fibroblast growth factor receptor 3
FGG	fibrinogen, gamma polypeptide	fibrinogen gamma chain
FGR	c-fgr,p55c-fgr; Gardner-Rasheed feline sarcoma viral (v-fgr) oncogene homolog,v-fgr feline Gardner-Rasheed sarcoma viral oncogene homolog,feline Gardner-Rasheed sarcoma viral oncogene homolog	FGR proto-oncogene, Src family tyrosine kinase
FKBP11	FKBP19; FK506 binding protein 11 (19 kDa),FK506 binding protein 11, 19 kDa,FK506 binding protein 11	FKBP prolyl isomerase 11
FLT1	vascular endothelial growth factor receptor 1,vascular permeability factor receptor; VEGFR1; fms-related tyrosine kinase 1 (vascular endothelial growth factor/vascular permeability factor receptor),fms-related tyrosine kinase 1,fms related tyrosine kinase 1	fms related receptor tyrosine kinase 1
FLT3LG	fms-related tyrosine kinase 3 ligand,fms related tyrosine kinase 3 ligand	fms related receptor tyrosine kinase 3 ligand
FN1	MSF,CIG,LETS,GFND2,FINC; migration-stimulating factor,cold-insoluble globulin	fibronectin 1
FOS	c-fos,AP-1; v-fos FBJ murine osteosarcoma viral oncogene homolog,FBJ murine osteosarcoma viral oncogene homolog	Fos proto-oncogene, AP-1 transcription factor subunit
FOXF1	FREAC1	forkhead box F1
FOXP3	JM2,XPID,AIID,PIDX,DIETER,SCURFIN; immune dysregulation, polyendocrinopathy, enteropathy, X-linked	forkhead box P3
FPR1	FPR,FMLP	formyl peptide receptor 1
FYB1	SLAP-130,FYB-120/130,ADAP; FYN-binding protein (FYB-120/130),FYN binding protein; adhesion and degranulation promoting adaptor protein	FYN binding protein 1
FYN	FYN oncogene related to SRC, FGR, YES; SYN,SLK,MGC45350	FYN proto-oncogene, Src family tyrosine kinase
FZD1	Wnt receptor,frizzled, Drosophila, homolog of, 1; frizzled (Drosophila) homolog 1,frizzled homolog 1 (Drosophila),frizzled 1, seven transmembrane spanning receptor,frizzled family receptor 1; DKFZp564G072	frizzled class receptor 1
FZD3	frizzled (Drosophila) homolog 3,frizzled homolog 3 (Drosophila),frizzled 3, seven transmembrane spanning receptor,frizzled family receptor 3	frizzled class receptor 3
FZD4	frizzled (Drosophila) homolog 4,exudative vitreoretinopathy 1,frizzled homolog 4 (Drosophila),frizzled 4, seven transmembrane spanning receptor,frizzled family receptor 4; CD344	frizzled class receptor 4
FZD5	frizzled (Drosophila) homolog 5,chromosome 2 open reading frame 31,frizzled homolog 5 (Drosophila),frizzled 5, seven transmembrane spanning receptor,frizzled family receptor 5; HFZ5,DKFZP434E2135	frizzled class receptor 5

FZD6	Hfz6; frizzled (Drosophila) homolog 6,frizzled homolog 6 (Drosophila),frizzled 6, seven transmembrane spanning receptor,frizzled family receptor 6	frizzled class receptor 6
FZD7	FzE3; frizzled (Drosophila) homolog 7,frizzled homolog 7 (Drosophila),frizzled 7, seven transmembrane spanning receptor,frizzled family receptor 7	frizzled class receptor 7
FZD8	frizzled (Drosophila) homolog 8,frizzled homolog 8 (Drosophila),frizzled 8, seven transmembrane spanning receptor,frizzled family receptor 8	frizzled class receptor 8
G6PC2	IGRP; islet specific glucose 6 phosphatase catalytic subunit related protein; glucose-6-phosphatase, catalytic, 2	glucose-6-phosphatase catalytic subunit 2
G6PD	G6PD1	glucose-6-phosphate dehydrogenase
GADD45B	myeloid differentiation primary response,growth arrest and DNA-damage-inducible beta; growth arrest and DNA-damage-inducible, beta; GADD45BETA,DKFZP566B133	growth arrest and DNA damage inducible beta
GAS6	AXSF,FLJ34709,DKFZp666G247; AXL stimulatory factor; AXL receptor tyrosine kinase ligand	growth arrest specific 6
GATA3	GATA-binding protein 3; HDR	GATA binding protein 3
GC	DBP,VDBP,hDBP; group-specific component (vitamin D binding protein),GC, vitamin D binding protein	GC vitamin D binding protein
GCG	glicentin-related polypeptide,glucagon-like peptide 1,glucagon-like peptide 2,preproglucagon; GLP1,GLP2,GRPP,GLP-1	glucagon
GDF10	BMP-3b	growth differentiation factor 10
GDF15	PLAB,MIC-1,PDF,MIC1,NAG-1,PTGFB; prostate differentiation factor,non-steroidal anti-inflammatory drug-activated gene-1,macrophage inhibitory cytokine-1	growth differentiation factor 15
GDF3		growth differentiation factor 3
GDF6	segmentation syndrome 1; BMP13,KFS,KFS1	growth differentiation factor 6
GDF9		growth differentiation factor 9
GDNF	astrocyte-derived trophic factor,glial cell line derived neurotrophic factor,glial derived neurotrophic factor; ATF1,ATF2,HFB1-GDNF	glial cell derived neurotrophic factor
GLUD1	GDH	glutamate dehydrogenase 1
GLUL	glutamate-ammonia ligase (glutamine synthase); glutamine synthetase	glutamate-ammonia ligase
GNLY	NKG5,LAG-2,D2S69E,TLA519; T-lymphocyte activation gene 519	granulysin
GPBAR1	BG37,GPCR,TGR5,M-BAR,GPCR19,GPR131,MGC40597	G protein-coupled bile acid receptor 1
GPER1	FEG-1,GPCR-Br,LERGU,LERGU2,DRY12,LyGPR,CEPR; G protein-coupled receptor 30	G protein-coupled estrogen receptor 1
GPNMB	transmembrane glycoprotein,glycoprotein NMB,glycoprotein nmb-like protein,osteoactivin,hematopoietic growth factor inducible neurokinin-1,glycoprotein nonmetastatic melanoma protein B; glycoprotein (transmembrane) nmb; NMB,HGFIN	glycoprotein nmb
GPR183	Epstein-Barr virus induced gene 2 (lymphocyte-specific G protein-coupled receptor); EBV-induced G-protein coupled receptor 2	G protein-coupled receptor 183
GPX1	selenoprotein GPX1	glutathione peroxidase 1
GPX3	glutathione peroxidase 3 (plasma); selenoprotein GPX3	glutathione peroxidase 3
GSN	DKFZp313L0718; gelsolin (amyloidosis, Finnish type); amyloidosis, Finnish type	gelsolin
GSTP1	GSTP	glutathione S-transferase pi 1
GZMA	CTL tryptase,Cytotoxic T-lymphocyte-associated serine esterase-3,Hanukah factor serine protease),granzyme 1; granzyme A (granzyme 1, cytotoxic T-lymphocyte-associated serine esterase 3)	granzyme A
GZMB	fragmentin 2,cytotoxic serine protease B,cathepsin G-like 1,T-cell serine protease 1-3E,granzyme 2,cytotoxic T-lymphocyte-associated serine esterase 1; CCPI,CGL-1,CSP-B,CGL1,CTSGL1,HLP,SECT; granzyme B (granzyme 2, cytotoxic T-lymphocyte-associated serine esterase 1)	granzyme B

GZMH	granzyme H (cathepsin G-like 2, protein h-CCPX); CGL-2,CCP-X,CTLA1,CSP-C; cathepsin G-like 2, protein h-CCPX	granzyme H
GZMK	tryptase II; granzyme K (serine protease, granzyme 3; tryptase II),granzyme K (granzyme 3; tryptase II); TRYP2,PRSS	granzyme K
H2AZ1	H2A histone family, member Z,H2A histone family member Z; H2A.Z	H2A.Z variant histone 1
H4C3	H4 histone family, member G,histone 1, H4c,histone cluster 1, H4c,histone cluster 1 H4 family member c; H4/g,dJ221C16.1	H4 clustered histone 3
HAVCR2	T-cell immunoglobulin mucin family member 3; Tim-3,TIM3,FLJ14428,TIMD3,CD366	hepatitis A virus cellular receptor 2
HBA1	HBA-T3; hemoglobin, alpha 1	hemoglobin subunit alpha 1
HBB	hemoglobin, beta; CD113t-C,beta-globin	hemoglobin subunit beta
HCAR2	HCA2,HM74A,PUMAG,Puma-g,NIACR1; G protein-coupled receptor 109A; niacin receptor 1	hydroxycarboxylic acid receptor 2
HCAR3	HCA3,HM74; G protein-coupled receptor 109B	hydroxycarboxylic acid receptor 3
HCK	hemopoietic cell kinase; JTK9	HCK proto-oncogene, Src family tyrosine kinase
HCST	DNAX-activation protein 10,kinase assoc pro of ~10kDa; phosphoinositide-3-kinase adaptor protein; DAP10,DKFZP586C1522,KAP10	hematopoietic cell signal transducer
HDAC1	HD1,GON-10,KDAC1	histone deacetylase 1
HDAC11		histone deacetylase 11
HDAC3	RPD3,HD3,RPD3-2,KDAC3	histone deacetylase 3
HDAC4	KIAA0288,HDAC-A,HDACA,HD4,HA6116,HDAC-4; brachydactyly-mental retardation syndrome	histone deacetylase 4
HDAC5	KIAA0600,NY-CO-9,FLJ90614	histone deacetylase 5
HGF	hepatopoietin A,fibroblast-derived tumor cytotoxic factor,scatter factor,lung fibroblast-derived mitogen; deafness, autosomal recessive 39,hepatocyte growth factor (hepapoietin A; scatter factor); SF,F-TCF,HGFB,HPTA	hepatocyte growth factor
HIF1A	MOP1,HIF-1alpha,PASD8,HIF1,bHLHe78; hypoxia inducible factor 1, alpha subunit (basic helix-loop-helix transcription factor)	hypoxia inducible factor 1 subunit alpha
HILPDA	FLJ21076,HIG-2,HIG2; hypoxia inducible gene 2; chromosome 7 open reading frame 68,hypoxia inducible lipid droplet-associated	hypoxia inducible lipid droplet associated
HLA-A		major histocompatibility complex, class I, A
HLA-B	ankylosing spondylitis	major histocompatibility complex, class I, B
HLA-C	psoriasis susceptibility 1	major histocompatibility complex, class I, C
HLA-DPA1		major histocompatibility complex, class II, DP alpha 1
HLA-DPB1		major histocompatibility complex, class II, DP beta 1
HLA-DQA1	CELIAC1	major histocompatibility complex, class II, DQ alpha 1
HLA-DQB1	IDDM1,CELIAC1	major histocompatibility complex, class II, DQ beta 1
HLA-DRA		major histocompatibility complex, class II, DR alpha
HLA-DRB1		major histocompatibility complex, class II, DR beta 1
HLA-DRB5		major histocompatibility complex, class II, DR beta 5
HLA-E		major histocompatibility complex, class I, E
HMGB2	high-mobility group (nonhistone chromosomal) protein 2,high-mobility group box 2	high mobility group box 2
HMGN2	high-mobility group (nonhistone chromosomal) protein 17,high-mobility group nucleosomal binding domain 2	high mobility group nucleosomal binding domain 2
HPGDS	glutathione S-transferase sigma; GSTS,PGDS,H-PGDS,PGD2,GSTS1-1,GSTS1	hematopoietic prostaglandin D synthase
HSD17B2	short chain dehydrogenase/reductase family 9C, member 2; hydroxysteroid (17-beta) dehydrogenase 2; HSD17,SDR9C2	hydroxysteroid 17-beta dehydrogenase 2
HSD3B2	short chain dehydrogenase/reductase family 11E, member 2; SDR11E2	hydroxy-delta-5-steroid dehydrogenase, 3 beta- and steroid delta-isomerase 2
HSP90AA1	heat shock 90kD protein 1, alpha,heat shock 90kDa protein 1, alpha,heat shock protein 90kDa alpha (cytosolic), class A member 1; Hsp89,Hsp90,FLJ31884,HSP90N	heat shock protein 90 alpha family class A member 1
HSP90AB1	heat shock 90kD protein 1, beta,heat shock 90kDa protein 1, beta,heat shock protein 90kDa alpha (cytosolic), class B member 1	heat shock protein 90 alpha family class B member 1

HSP90B1	tumor rejection antigen (gp96) 1,heat shock protein 90kDa beta (Grp94), member 1; GP96,GRP94; endoplasmin	heat shock protein 90 beta family member 1
HSPA1A	heat shock 70kD protein 1A,heat shock 70kDa protein 1A; HSP70-1	heat shock protein family A (Hsp70) member 1A
HSPA1B	HSP70-2; heat shock 70kD protein 1B,heat shock 70kDa protein 1B	heat shock protein family A (Hsp70) member 1B
HSPB1	HSP27,HSP28,Hs.76067,Hsp25,CMT2F; heat shock 27kD protein 1,heat shock 27kDa protein 1	heat shock protein family B (small) member 1
HTT	IT15; huntingtin (Huntington disease)	huntingtin
IAPP	AMYLIN,DAP,IAP; amylin	islet amyloid polypeptide
ICAM1	BB2,CD54; human rhinovirus receptor	intercellular adhesion molecule 1
ICAM2	CD102	intercellular adhesion molecule 2
ICAM3	CDW50,ICAM-R,CD50	intercellular adhesion molecule 3
ICOS	activation-inducible lymphocyte immunomediatory molecule; AILIM,CD278; inducible T-cell costimulator	inducible T cell costimulator
ICOSLG	KIAA0653,GL50,B7-H2,B7RP-1,B7H2,B7RP1,ICOS-L,CD275,B7h; inducible T-cell costimulator ligand; B7-related protein 1,B7 homologue 2,B7 homolog 2	inducible T cell costimulator ligand
IDO1	indoleamine-pyrrole 2,3 dioxygenase	indoleamine 2,3-dioxygenase 1
IER3	IEX-1,DIF-2,PRG1,IEX-1L	immediate early response 3
IFI27	P27,FAM14D,ISG12	interferon alpha inducible protein 27
IFIH1	MDA-5,Hlcd,MDA5,IDDM19; helicard,melanoma differentiation-associated gene 5	interferon induced with helicase C domain 1
IFIT1	GARG-16; interferon-induced protein with tetratricopeptide repeats 1	interferon induced protein with tetratricopeptide repeats 1
IFITM1	interferon-induced transmembrane protein 1,dispanin subfamily A member 2a; 9-27,CD225,DSPA2a; interferon induced transmembrane protein 1 (9-27)	interferon induced transmembrane protein 1
IFITM3	dispanin subfamily A member 2b; 1-8U,DSPA2b; interferon induced transmembrane protein 3 (1-8U)	interferon induced transmembrane protein 3
IFNA1	IFNA@,IFL,IFN,IFN-ALPHA,IFNA13,IFN-alphaD; IFN-alpha 1b,interferon alpha 1b	interferon alpha 1
IFNAR1	IFRC; IFNalpha/beta receptor 1,type I interferon receptor 1,interferon alpha/beta receptor 1; interferon (alpha, beta and omega) receptor 1	interferon alpha and beta receptor subunit 1
IFNAR2	interferon alpha/beta receptor 2,IFNalpha/beta receptor subunit 2,type I interferon receptor 2; interferon (alpha, beta and omega) receptor 2	interferon alpha and beta receptor subunit 2
IFNB1	IFB,IFF; interferon, beta 1, fibroblast	interferon beta 1
IFNG		interferon gamma
IFNGR1	CD119	interferon gamma receptor 1
IFNGR2	interferon gamma receptor 2 (interferon gamma transducer 1); AF-1	interferon gamma receptor 2
IFNL2	interleukin 28A,interleukin 28A (interferon, lambda 2); IL-28A	interferon lambda 2
IFNL3	interleukin 28B,interleukin 28B (interferon, lambda 3); IL-28B,IL28C	interferon lambda 3
IGF1	IGF1A,IGFI,IGF-I,IGF; somatomedin C; insulin-like growth factor 1 (somatomedin C)	insulin like growth factor 1
IGF1R	JTK13,CD221,IGFIR,MGC18216,IGFR; insulin-like growth factor 1 receptor	insulin like growth factor 1 receptor
IGF2	FLJ44734,IGF-II; somatomedin A,preptin; chromosome 11 open reading frame 43,insulin-like growth factor 2	insulin like growth factor 2
IGF2R	CD222,MPRI,MPR1,CIMPR,M6P-R,CI-M6PR,CI-MPR,MPR300; insulin-like growth factor 2 receptor; cation-independent mannose-6 phosphate receptor	insulin like growth factor 2 receptor
IGFBP3	insulin-like growth factor binding protein 3; growth hormone-dependent binding protein,acid stable subunit of the 140 K IGF complex,binding protein 53,binding protein 29,IGF-binding protein 3; IBP3,BP-53	insulin like growth factor binding protein 3
IGFBP5	insulin-like growth factor binding protein 5	insulin like growth factor binding protein 5
IGFBP6	insulin-like growth factor binding protein 6	insulin like growth factor binding protein 6
IGFBP7	insulin-like growth factor binding protein 7; MAC25,IGFBP-7,PSF,FSTL2	insulin like growth factor binding protein 7
IGHA1		immunoglobulin heavy constant alpha 1

IGHD	FLJ00382,FLJ46727,MGC29633; immunoglobulin delta,constant region of heavy chain of IgD	immunoglobulin heavy constant delta
IGHG1		immunoglobulin heavy constant gamma 1 (G1m marker)
IGHG2		immunoglobulin heavy constant gamma 2 (G2m marker)
IGHM		immunoglobulin heavy constant mu
IGKC		immunoglobulin kappa constant
IL10	CSIF,TGIF,IL10A,IL-10; cytokine synthesis inhibitory factor,T-cell growth inhibitory factor	interleukin 10
IL10RA	interleukin 10 receptor, alpha; HIL-10R,CDW210A,CD210a,CD210	interleukin 10 receptor subunit alpha
IL10RB	interleukin 10 receptor, beta; CRF2-4,CDW210B,IL-10R2	interleukin 10 receptor subunit beta
IL11	IL-11,AGIF; adipogenesis inhibitory factor,oprelvekin	interleukin 11
IL11RA	interleukin 11 receptor, alpha	interleukin 11 receptor subunit alpha
IL12A	interleukin 12A (natural killer cell stimulatory factor 1, cytotoxic lymphocyte maturation factor 1, p35); CLMF,IL-12A,p35,NFSK; natural killer cell stimulatory factor 1, 35 kD subunit,cytotoxic lymphocyte maturation factor 1, p35,interleukin 12, p35,IL-12, subunit p35,NF cell stimulatory factor chain 1,interleukin-12 alpha chain,IL35 subunit	interleukin 12A
IL12B	CLMF,IL-12B,NKSF,CLMF2; interleukin 12B (natural killer cell stimulatory factor 2, cytotoxic lymphocyte maturation factor 2, p40); natural killer cell stimulatory factor-2,cytotoxic lymphocyte maturation factor 2, p40,interleukin 12, p40,natural killer cell stimulatory factor, 40 kD subunit,interleukin-12 beta chain,IL12, subunit p40	interleukin 12B
IL12RB1	interleukin 12 receptor, beta 1; CD212	interleukin 12 receptor subunit beta 1
IL12RB2	interleukin 12 receptor, beta 2	interleukin 12 receptor subunit beta 2
IL13RA1	interleukin 13 receptor, alpha 1; IL-13Ra,NR4,CD213a1; IL13 receptor alpha-1 chain,CD213a1 antigen	interleukin 13 receptor subunit alpha 1
IL15	IL-15,MGC9721	interleukin 15
IL15RA	CD215,IL-15RA; interleukin 15 receptor, alpha	interleukin 15 receptor subunit alpha
IL16	interleukin 16 (lymphocyte chemoattractant factor); prointerleukin 16,lymphocyte chemoattractant factor; LCF,IL-16,prIL-16,HsT19289,FLJ42735,FLJ16806	interleukin 16
IL17A	interleukin 17 (cytotoxic T-lymphocyte-associated serine esterase 8); IL-17A,IL-17; cytotoxic T-lymphocyte-associated protein 8	interleukin 17A
IL17B	IL-17B,ZCYTO7,IL-20,MGC138900,MGC138901,NIRF; neuronal interleukin-17-related factor	interleukin 17B
IL17D	interleukin 27; IL-27,IL-17D,IL27,FLJ30846	interleukin 17D
IL17RA	hIL-17R,IL-17RA,CDw217,CD217; interleukin 17 receptor	interleukin 17 receptor A
IL17RB	IL17RH1,EVI27,CRL4; interleukin 17B receptor	interleukin 17 receptor B
IL17RE	FLJ23658	interleukin 17 receptor E
IL18	IGIF,IL1F4,IL-1g,IL-18; interferon-gamma-inducing factor; interleukin 18 (interferon-gamma-inducing factor)	interleukin 18
IL18R1	IL1RRP,IL-1Rrp,CD218a	interleukin 18 receptor 1
IL1A	IL1F1,IL-1A,IL1-ALPHA; preinterleukin 1 alpha,hematopoietin-1,pro-interleukin-1-alpha	interleukin 1 alpha
IL1B	IL1F2,IL-1B,IL1-BETA	interleukin 1 beta
IL1R1	interleukin-1 receptor, type I; interleukin 1 receptor, type I; D2S1473,CD121A	interleukin 1 receptor type 1
IL1R2	CD121b; interleukin 1 receptor, type II	interleukin 1 receptor type 2
IL1RAP	IL-1RAcP,IL1R3,C3orf13	interleukin 1 receptor accessory protein
IL1RL1	ST2,FIT-1,ST2L,ST2V,DER4,T1,IL33R; homolog of mouse growth stimulation-expressed	interleukin 1 receptor like 1
IL1RN	interleukin-1 receptor antagonist protein,intracellular interleukin-1 receptor antagonist; IL1RA,ICIL-1RA,IL1F3,IRAP,IL-1RN,MGC10430	interleukin 1 receptor antagonist
IL2	IL-2,TCGF; T cell growth factor	interleukin 2
IL20	ZCYTO10,IL10D,IL-20	interleukin 20
IL20RA	interleukin 20 receptor, alpha,interleukin 20 receptor alpha subunit; ZCYTOR7,IL-20R1	interleukin 20 receptor subunit alpha
IL22RA1	CRF2-9; interleukin 22 receptor,interleukin 22 receptor, alpha 1	interleukin 22 receptor subunit alpha 1

IL23A	interleukin 23, alpha subunit p19; interleukin-six, G-CSF related factor; SGRF,IL23P19,IL-23,IL-23A,P19	interleukin 23 subunit alpha
IL24	mda-7,IL10B,Mob-5,C49A,FISP,IL-24; melanoma differentiation association protein 7,suppression of tumorigenicity 16 (melanoma differentiation),IL-4-induced secreted protein	interleukin 24
IL27RA	T-cell cytokine receptor type 1; WSX-1,TCCR,CRL1,WSX1,zcytor1,IL-27R; interleukin 27 receptor, alpha	interleukin 27 receptor subunit alpha
IL2RA	insulin-dependent diabetes mellitus 10,interleukin 2 receptor, alpha; CD25	interleukin 2 receptor subunit alpha
IL2RB	interleukin 15 receptor, beta,interleukin 2 receptor, beta; CD122	interleukin 2 receptor subunit beta
IL2RG	severe combined immunodeficiency,combined immunodeficiency, X-linked,interleukin 2 receptor, gamma; CD132	interleukin 2 receptor subunit gamma
IL32	NK4,TAIF,TAIFb,TAIFd; natural killer cell transcript 4	interleukin 32
IL33	DVS27-related protein,nuclear factor for high endothelial venules,interleukin-1 family, member 11; chromosome 9 open reading frame 26 (NF-HEV); DVS27,DKFZp586H0523,NF-HEV,IL1F11	interleukin 33
IL34	MGC34647,IL-34; chromosome 16 open reading frame 77	interleukin 34
IL36G	IL-1H1,IL-1RP2,IL-1F9,IL1H1,IL1E; interleukin 1 family, member 9; interleukin-1 homolog 1,interleukin 1-related protein 2,interleukin-1 epsilon	interleukin 36 gamma
IL3RA	interleukin 3 receptor, alpha (low affinity); CD123	interleukin 3 receptor subunit alpha
IL4R	interleukin 13 receptor; CD124	interleukin 4 receptor
IL6	interferon, beta 2; IL-6,BSF2,HGF,HSF; interleukin 6 (interferon, beta 2)	interleukin 6
IL6R	membrane glycoprotein 80,interleukin 6 receptor subunit alpha; CD126,IL-6R,IL-1Ra,IL6RA,gp80	interleukin 6 receptor
IL6ST	GP130,CD130,sGP130,IL-6RB; gp130, oncostatin M receptor,Interleukin-6 receptor subunit beta,membrane glycoprotein 130; interleukin 6 signal transducer (gp130, oncostatin M receptor),interleukin 6 signal transducer	interleukin 6 cytokine family signal transducer
IL7	IL-7	interleukin 7
IL7R	CD127,IL7RA	interleukin 7 receptor
INHA	inhibin, alpha	inhibin subunit alpha
INHBA	inhibin, beta A (activin A, activin AB alpha polypeptide),inhibin, beta A	inhibin subunit beta A
INHBB	inhibin, beta B (activin AB beta polypeptide),inhibin, beta B	inhibin subunit beta B
INS	insulin-dependent diabetes mellitus 2	insulin
INSR	CD220	insulin receptor
IRF3		interferon regulatory factor 3
IRF4	LSIRF	interferon regulatory factor 4
ITGA1	VLA1,CD49a; integrin, alpha 1	integrin subunit alpha 1
ITGA2	alpha 2 subunit of VLA-2 receptor; CD49b; integrin, alpha 2 (CD49B, alpha 2 subunit of VLA-2 receptor)	integrin subunit alpha 2
ITGA3	CD49c,VLA3a,VCA-2,GAP-B3; antigen identified by monoclonal antibody J143,integrin, alpha 3 (antigen CD49C, alpha 3 subunit of VLA-3 receptor); alpha 3 subunit of VLA-3 receptor,antigen CD49C	integrin subunit alpha 3
ITGA5	fibronectin receptor, alpha polypeptide; integrin, alpha 5 (fibronectin receptor, alpha polypeptide); CD49e	integrin subunit alpha 5
ITGA6	CD49f; integrin, alpha 6	integrin subunit alpha 6
ITGA9	integrin, alpha 9; integrin, alpha 4-like; RLC,ITGA4L,ALPHA-RLC	integrin subunit alpha 9
ITGAE	integrin, alpha E (antigen CD103, human mucosal lymphocyte antigen 1; alpha polypeptide); CD103,HUMINAE; antigen CD103,human mucosal lymphocyte antigen 1, alpha polypeptide	integrin subunit alpha E

ITGAL	antigen CD11A (p180),lymphocyte function-associated antigen 1, alpha polypeptide; integrin, alpha L (antigen CD11A (p180), lymphocyte function-associated antigen 1; alpha polypeptide); LFA-1	integrin subunit alpha L
ITGAM	complement component 3 receptor 3 subunit; integrin, alpha M (complement component receptor 3, alpha; also known as CD11b (p170), macrophage antigen alpha polypeptide),integrin, alpha M (complement component 3 receptor 3 subunit); MAC-1,CD11b	integrin subunit alpha M
ITGAV	CD51; antigen identified by monoclonal antibody L230,vitronectin receptor,integrin, alpha V (vitronectin receptor, alpha polypeptide, antigen CD51),integrin, alpha V	integrin subunit alpha V
ITGAX	integrin, alpha X (antigen CD11C (p150), alpha polypeptide),integrin, alpha X (complement component 3 receptor 4 subunit); complement component 3 receptor 4 subunit; CD11c	integrin subunit alpha X
ITGB1	integrin, beta 1 (fibronectin receptor, beta polypeptide, antigen CD29 includes MDF2, MSK12); CD29,GPIIA	integrin subunit beta 1
ITGB2	integrin, beta 2 (antigen CD18 (p95), lymphocyte function-associated antigen 1; macrophage antigen 1 (mac-1) beta subunit),integrin, beta 2 (complement component 3 receptor 3 and 4 subunit); LFA-1,MAC-1; complement component 3 receptor 3 and 4 subunit	integrin subunit beta 2
ITGB4	integrin, beta 4; CD104	integrin subunit beta 4
ITGB5	integrin, beta 5	integrin subunit beta 5
ITGB6	integrin, beta 6	integrin subunit beta 6
ITGB8	integrin, beta 8	integrin subunit beta 8
ITK	IL2-inducible T-cell kinase,IL2 inducible T-cell kinase; EMT,PSCTK2,LYK	IL2 inducible T cell kinase
ITM2A	BRICD2A,E25A; BRICHOS domain containing 2A	integral membrane protein 2A
JAG1	AHD,AWS,HJ1,CD339; Alagille syndrome,jagged 1	jagged canonical Notch ligand 1
JAK1	JAK1A,JTK3	Janus kinase 1
JAK2	JTK10	Janus kinase 2
JCHAIN	immunoglobulin J polypeptide, linker protein for immunoglobulin alpha and mu polypeptides; immunoglobulin J chain,IgJ chain,J chain; IGCJ,JCH	joining chain of multimeric IgA and IgM
JUN	c-Jun,AP-1; v-jun avian sarcoma virus 17 oncogene homolog,jun oncogene	Jun proto-oncogene, AP-1 transcription factor subunit
JUNB		JunB proto-oncogene, AP-1 transcription factor subunit
KDR	vascular endothelial growth factor receptor 2,fetal liver kinase 1; FLK1,VEGFR,VEGFR2,CD309; kinase insert domain receptor (a type III receptor tyrosine kinase)	kinase insert domain receptor
KIT	mast/stem cell growth factor receptor Kit; CD117,SCFR,C-Kit; piebald trait,v-kit Hardy-Zuckerman 4 feline sarcoma viral oncogene homolog	KIT proto-oncogene, receptor tyrosine kinase
KITLG	mast cell growth factor,stem cell factor,steel factor,familial progressive hyperpigmentation 2; SCF,SF,Kitl,KL-1,FPH2,SLF,DFNA69	KIT ligand
KLF2	lung Kruppel-like factor; LKLF; Kruppel-like factor 2 (lung)	Kruppel like factor 2
KLK3	PSA; kallikrein 3, (prostate specific antigen)	kallikrein related peptidase 3
KLRB1	killer cell lectin-like receptor subfamily B, member 1; CD161,NKR-P1,NKR-P1A,hNKR-P1A,CLEC5B; natural killer cell surface protein P1A	killer cell lectin like receptor B1
KLRK1	NKG2D,KLR,NKG2-D,CD314; DNA segment on chromosome 12 (unique) 2489 expressed sequence,killer cell lectin-like receptor subfamily K, member 1	killer cell lectin like receptor K1
KRAS	KRAS1,K-Ras4B; v-Ki-ras2 Kirsten rat sarcoma 2 viral oncogene homolog,v-Ki-ras2 Kirsten rat sarcoma viral oncogene homolog,Kirsten rat sarcoma viral oncogene homolog	KRAS proto-oncogene, GTPase
KRT1	epidermolytic hyperkeratosis 1,keratin 1, type II; KRT1A	keratin 1
KRT10	K10,CK10; keratosis palmaris et plantaris,keratin 10, type I; cytokeratin 10,epidermolytic hyperkeratosis	keratin 10

KRT13	K13,CK13,MGC3781,MGC161462; keratin 13, type I; keratin, type I cytoskeletal 13,cytokeratin 13	keratin 13
KRT14	keratin 14 (epidermolysis bullosa simplex, Dowling-Meara, Koebner),keratin 14, type I; epidermolysis bullosa simplex, Dowling-Meara, Koebner	keratin 14
KRT15	keratin-15, basic,keratin-15, beta,type I cytoskeletal 15,cytokeratin 15; K15,CK15,K1CO; keratin 15, type I	keratin 15
KRT16	NEPPK; keratin 16, type I; focal non-epidermolytic palmoplantar keratoderma	keratin 16
KRT17	keratin 17, type I	keratin 17
KRT18	keratin 18, type I	keratin 18
KRT19	keratin 19, type I; keratin, type I cytoskeletal 19,keratin, type I, 40-kd,cytokeratin 19,40-kDa keratin intermediate filament; K19,CK19,K1CS,MGC15366	keratin 19
KRT20	keratin 20, type I; CK20,K20,MGC35423	keratin 20
KRT23	keratin 23 (histone deacetylase inducible),keratin 23, type I; K23,DKFZP434G032,HAIK1,CK23,MGC26158	keratin 23
KRT24	keratin 24, type I; FLJ20261,MGC138169,MGC138173	keratin 24
KRT4	CK4,K4; CYK4; cytokeratin 4,keratin, type II cytoskeletal 4	keratin 4
KRT5	epidermolysis bullosa simplex 2 Dowling-Meara/Kobner/Weber-Cockayne types,keratin 5 (epidermolysis bullosa simplex, Dowling-Meara/Kobner/Weber-Cockayne types),keratin 5, type II; KRT5A	keratin 5
KRT6A	CK6C,K6C,CK6D,K6D; keratin 6C,keratin 6D,keratin 6A, type II	keratin 6A
KRT6B	keratin-like 1 (a type II keratin sequence),keratin 6B, type II	keratin 6B
KRT6C	keratin 6E,keratin 6C, type II	keratin 6C
KRT7	keratin, type II cytoskeletal 7,cytokeratin 7,sarcolectin,keratin, 55K type II cytoskeletal; keratin 7, type II; K7,CK7,K2C7,SCL	keratin 7
KRT8	keratin 8, type II; CARD2,K8,CK8,CYK8,K2C8,KO	keratin 8
KRT80	keratin 80, type II; KB20	keratin 80
KRT86	keratin, hair, basic, 6 (monilethrix),keratin 86, type II; hard keratin type II 6; MNX,Hb6	keratin 86
LAG3	lymphocyte-activation gene 3; CD223	lymphocyte activating 3
LAIR1	CD305,LAIR-1; leukocyte-associated Ig-like receptor 1,leukocyte-associated immunoglobulin-like receptor 1	leukocyte associated immunoglobulin like receptor 1
LAMP2	lysosomal-associated membrane protein 2; CD107b	lysosomal associated membrane protein 2
LAMP3	LAMP,TSC403,DC-LAMP,DCLAMP,CD208; lysosomal-associated membrane protein 3	lysosomal associated membrane protein 3
LCN2	NGAL,24p3; oncogene 24p3,neutrophil gelatinase-associated lipocalin,siderocalin; lipocalin 2 (oncogene 24p3)	lipocalin 2
LDLR	familial hypercholesterolemia; LDLCQ2	low density lipoprotein receptor
LEFTY1	LEFTYB; left-right determination, factor B	left-right determination factor 1
LEFTY2	endometrial bleeding associated factor (left-right determination, factor A; transforming growth factor beta superfamily); transforming growth factor, beta-4 (endometrial bleeding-associated factor; LEFTY A); LEFTA,LEFTYA	left-right determination factor 2
LEP	leptin (murine obesity homolog),leptin (obesity homolog, mouse)	leptin
LGALS1	lectin, galactoside-binding, soluble, 1; GBP	galectin 1
LGALS3	lectin, galactoside-binding, soluble, 3; MAC-2,GALIG; advanced glycation end-product receptor 3	galectin 3
LGALS3BP	MAC-2-BP,90K,BTBD17B,TANGO10B,M2BP,gp90,CyCAP; L3 antigen,Mac-2-binding protein,serum protein 90K,transport and golgi organization 10 homolog B (Drosophila); lectin, galactoside-binding, soluble, 3 binding protein	galectin 3 binding protein
LGALS9	LGALS9A; lectin, galactoside-binding, soluble, 9	galectin 9

LIF	differentiation inhibitory activity,differentiation-inducing factor,hepatocyte-stimulating factor III,cholinergic differentiation factor,human interleukin in DA cells; CDF,DIA,HILDA; leukemia inhibitory factor,LIF, interleukin 6 family cytokine	LIF interleukin 6 family cytokine
LIFR	CD118; leukemia inhibitory factor receptor,leukemia inhibitory factor receptor alpha,LIF receptor alpha	LIF receptor subunit alpha
LINC02446		long intergenic non-protein coding RNA 2446
LMNA	cardiomyopathy, dilated 1A (autosomal dominant),limb girdle muscular dystrophy 1B (autosomal dominant),progeria 1 (Hutchinson-Gilford type),lamin A/C-like 1; HGPS,MADA; mandibuloacral dysplasia type A	lamin A/C
LPAR5	KPG_010,LPA5; G protein-coupled receptor 92	lysophosphatidic acid receptor 5
LTB	TNF superfamily member 3; p33,TNFSF3	lymphotoxin beta
LTBR	TNFR superfamily, member 3; TNFCR,TNFR-RP,TNFR2-RP,TNF-R-III,TNFRSF3	lymphotoxin beta receptor
LTF	HLF2	lactotransferrin
LUM	SLRR2D; lumican proteoglycan	lumican
LY6D	lymphocyte antigen 6 complex, locus D; E48	lymphocyte antigen 6 family member D
LY75	DEC-205,CLEC13B,CD205	lymphocyte antigen 75
LYN	v-yes-1 Yamaguchi sarcoma viral related oncogene homolog; JTK8	LYN proto-oncogene, Src family tyrosine kinase
LYZ	renal amyloidosis; lysozyme (renal amyloidosis)	lysozyme
MAF	c-MAF; v-maf avian musculoaponeurotic fibrosarcoma oncogene homolog	MAF bZIP transcription factor
MALAT1	PRO1073,MALAT-1,NCRNA00047,HCN,NEAT2,LINC00047,mascRNA; metastasis associated lung adenocarcinoma transcript 1 (non-protein coding); metastasis associated in lung adenocarcinoma transcript 1,non-protein coding RNA 47,hepcarcin,nuclear enriched abundant transcript 2,nuclear paraspeckle assembly transcript 2 (non-protein coding),long intergenic non-protein coding RNA 47	metastasis associated lung adenocarcinoma transcript 1
MAML2	mastermind (Drosophila)-like 2,mastermind-like 2 (Drosophila); KIAA1819,MAM3	mastermind like transcriptional coactivator 2
MAP1LC3B	ATG8F	microtubule associated protein 1 light chain 3 beta
MAPK13	SAPK4,p38delta	mitogen-activated protein kinase 13
MAPK14	PRKM14,p38,Mxi2,PRKM15; p38 MAP kinase	mitogen-activated protein kinase 14
MARCO	scavenger receptor class A, member 2; SCARA2,SR-A6	macrophage receptor with collagenous structure
MECOM	myelodysplasia syndrome 1,ecotropic viral integration site 1; PR domain 3; MDS1-EVI1,PRDM3,KMT8E	MDS1 and EVI1 complex locus
MEG3	non-protein coding RNA 23,long intergenic non-protein coding RNA 23; maternally expressed 3,maternally expressed 3 (non-protein coding); GTL2,NCRNA00023,LINC00023,onco-lncRNA-83	maternally expressed 3
MERTK	c-mer proto-oncogene tyrosine kinase; mer,RP38,c-Eyk,Tyro12	MER proto-oncogene, tyrosine kinase
MET	hepatocyte growth factor receptor; met proto-oncogene; HGFR,RCCP2,DFNB97	MET proto-oncogene, receptor tyrosine kinase
MGP		matrix Gla protein
MIF	macrophage migration inhibitory factor (glycosylation-inhibiting factor); glycosylation-inhibiting factor,phenylpyruvate tautomerase; GIF	macrophage migration inhibitory factor
MKI67	MIB-1,PPP1R105; antigen identified by monoclonal antibody Ki-67; protein phosphatase 1, regulatory subunit 105	marker of proliferation Ki-67
MMP1	matrix metalloproteinase 1 (interstitial collagenase); interstitial collagenase	matrix metallopeptidase 1
MMP10	matrix metalloproteinase 10 (stromelysin 2),stromelysin 2	matrix metallopeptidase 10
MMP12	matrix metalloproteinase 12 (macrophage elastase); HME; macrophage elastase	matrix metallopeptidase 12
MMP14	matrix metalloproteinase 14 (membrane-inserted),matrix metallopeptidase 14 (membrane-inserted); MT1-MMP; membrane type 1 metalloprotease,membrane type 1-matrix metalloproteinase	matrix metallopeptidase 14

MMP16	MT3-MMP,DKFZp761D112; matrix metalloproteinase 16 (membrane-inserted),chromosome 8 open reading frame 57,matrix metallopeptidase 16 (membrane-inserted)	matrix metallopeptidase 16
MMP19	matrix metalloproteinase 19; RASI-1	matrix metallopeptidase 19
MMP2	matrix metalloproteinase 2 (gelatinase A, 72kDa gelatinase, 72kDa type IV collagenase); TBE-1	matrix metallopeptidase 2
MMP3	matrix metalloproteinase 3 (stromelysin 1, progelatinase),stromelysin 1	matrix metallopeptidase 3
MMP7	PUMP-1; matrix metalloproteinase 7 (matrilysin, uterine); matrilysin	matrix metallopeptidase 7
MMP8	matrix metalloproteinase 8 (neutrophil collagenase)	matrix metallopeptidase 8
MMP9	matrix metalloproteinase 9 (gelatinase B, 92kDa gelatinase, 92kDa type IV collagenase)	matrix metallopeptidase 9
MPO		myeloperoxidase
MRC1	macrophage mannose receptor; mannose receptor, C type 1-like 1,mannose receptor, C type 1; CLEC13D,CD206,bA541I19.1,CLEC13DL	mannose receptor C-type 1
MRC2	mannose receptor, C type 2; KIAA0709,ENDO180,CLEC13E,CD280; C-type lectin domain family 13 member E,endocytic receptor 180	mannose receptor C type 2
MS4A1	membrane-spanning 4-domains, subfamily A, member 1; B1,Bp35,MS4A2,FMC7	membrane spanning 4-domains A1
MS4A4A	membrane-spanning 4-domains, subfamily A, member 4,membrane-spanning 4-domains, subfamily A, member 4A; CD20L1,MS4A7	membrane spanning 4-domains A4A
MSMB	beta-microseminoprotein; microseminoprotein, beta-; PSP-94,PSP57,PSP94,IGBF,MSP,MSPB,PN44,PRPS,PSP	microseminoprotein beta
MST1R	CDw136,CD136; c-met-related tyrosine kinase; PTK8 protein tyrosine kinase 8,S13 erythroblastosis (avian) oncogene homolog,S13 avian erythroblastosis oncogene homolog	macrophage stimulating 1 receptor
MT1X	MT-1l	metallothionein 1X
MT2A		metallothionein 2A
MTOR	FK506 binding protein 12-rapamycin associated protein 2,rapamycin target protein,FKBP12-rapamycin complex-associated protein 1,FKBP-rapamycin associated protein,rapamycin associated protein FRAP2,dJ576K7.1 (FK506 binding protein 12-rapamycin associated protein 1),rapamycin and FKBP12 target 1,mammalian target of rapamycin; RAFT1,RAPT1,FLJ44809; FK506 binding protein 12-rapamycin associated protein 1,mechanistic target of rapamycin (serine/threonine kinase),mechanistic target of rapamycin	mechanistic target of rapamycin kinase
MTRNR2L1	humanin-like 1	MT-RNR2 like 1
MX1	IFI-78K,MxA,lncMX1-215; interferon-inducible protein p78; myxovirus (influenza) resistance 1, homolog of murine (interferon-inducible protein p78),myxovirus (influenza virus) resistance 1, interferon-inducible protein p78 (mouse)	MX dynamin like GTPase 1
MXRA8	limitrin; matrix-remodelling associated 8; DKFZp586E2023	matrix remodeling associated 8
MYC	c-Myc,bHLHe39,MYCC; v-myc avian myelocytomatosis viral oncogene homolog	MYC proto-oncogene, bHLH transcription factor
MYH11	myosin, heavy polypeptide 11, smooth muscle; SMMHC,SMHC	myosin heavy chain 11
MYL9	myosin, light polypeptide 9, regulatory,myosin, light chain 9, regulatory; myosin regulatory light chain 2, smooth muscle isoform,myosin regulatory light chain 1; MYRL2,MLC2,LC20,MRLC1	myosin light chain 9
MZB1	plasma cell-induced ER protein 1,proapoptotic caspase adaptor protein,mesenteric oestrogen-dependent adipose gene- 7; marginal zone B and B1 cell-specific protein; PACAP,MGC29506,HSPC190,pERp1,MEDA-7	marginal zone B and B1 cell specific protein
MZT2A	family with sequence similarity 128, member A; MOZART2A; mitotic-spindle organizing protein associated with a ring of gamma-tubulin 2A	mitotic spindle organizing protein 2A

NANOG	FLJ12581,FLJ40451	Nanog homeobox
NCR1	lymphocyte antigen 94 (mouse) homolog (activating NK-receptor; NK-p46); NK-p46,NKP46,CD335	natural cytotoxicity triggering receptor 1
NDRG1	DRG1,RTP,TDD5,NDR1	N-myc downstream regulated 1
NEAT1	non-protein coding RNA 84,nuclear paraspeckle assembly transcript 1 (non-protein coding); trophoblast derived non-protein coding RNA,nuclear enriched abundant transcript 1,long intergenic non-protein coding RNA 84,virus inducible non-coding RNA; TncRNA,MENepsilon/beta,LINC00084,VINC	nuclear paraspeckle assembly transcript 1
NFKB1	nuclear factor of kappa light polypeptide gene enhancer in B-cells 1; Nuclear factor NF-kappa-B p105 subunit; KBF1,p105,NFKB-p50,p50,NF-kappaB,NFkappaB,NF-kB1	nuclear factor kappa B subunit 1
NFKBIA	IKBA,MAD-3,IkappaBalpha; NF-kappa-B inhibitor alpha; nuclear factor of kappa light polypeptide gene enhancer in B-cells inhibitor, alpha	NFKB inhibitor alpha
NGFR	nerve growth factor receptor (TNFR superfamily, member 16); TNFRSF16,CD271,p75NTR; low affinity nerve growth factor receptor,TNFR superfamily, member 16	nerve growth factor receptor
NKG7	natural killer cell group 7 sequence; GIG1,GMP-17; granule membrane protein 17	natural killer cell granule protein 7
NLRC4	nucleotide-binding oligomerization domain, leucine rich repeat and CARD domain containing 4,NOD-like receptor C4; caspase recruitment domain family, member 12; CLAN1,ipaf,CLANA,CLANB,CLANC,CLAND,CLR2.1,CLAN	NLR family CARD domain containing 4
NLRC5	NOD27,CLR16.1,FLJ21709; nucleotide-binding oligomerization domain, leucine rich repeat and CARD domain containing 5,NOD-like receptor C5	NLR family CARD domain containing 5
NLRP1	NACHT, leucine rich repeat and PYD (pyrin domain) containing 1,systemic lupus erythematosus, vitiligo-related 1; nucleotide-binding oligomerization domain, leucine rich repeat and pyrin domain containing 1; KIAA0926,DKFZp586O1822,CARD7,NAC,CLR17.1,DEFCAP,VAMAS1	NLR family pyrin domain containing 1
NLRP2	FLJ20510,PYPAF2,NBS1,PAN1,CLR19.9; nucleotide-binding oligomerization domain, leucine rich repeat and pyrin domain containing 2; NACHT, leucine rich repeat and PYD containing 2	NLR family pyrin domain containing 2
NLRP3	cold autoinflammatory syndrome 1,deafness, autosomal dominant 34; AGTAVPRL,AII,AVP,FCAS,FCU,NALP3,PYPAF1,MWS,CLR1.1; Cryopyrin,nucleotide-binding oligomerization domain, leucine rich repeat and pyrin domain containing 3	NLR family pyrin domain containing 3
NOD2	caspase recruitment domain family, member 15; nucleotide-binding oligomerization domain, leucine rich repeat and CARD domain containing 2,NOD-like receptor C2,NLR family, CARD domain containing 2; BLAU,CD,PSORAS1,CLR16.3,NLRC2	nucleotide binding oligomerization domain containing 2
NOSIP	CGI-25	nitric oxide synthase interacting protein
NOTCH1	Notch (Drosophila) homolog 1 (translocation-associated),Notch homolog 1, translocation-associated (Drosophila),notch 1	notch receptor 1
NOTCH2	Notch (Drosophila) homolog 2,Notch homolog 2 (Drosophila),notch 2	notch receptor 2
NOTCH3	CASIL; Notch (Drosophila) homolog 3,Notch homolog 3 (Drosophila),notch 3	notch receptor 3
NPPB	natriuretic peptide precursor B	natriuretic peptide B
NPPC	natriuretic peptide precursor C; CNP	natriuretic peptide C
NPR1	guanylate cyclase A; GUCY2A,ANPa; atrionatriuretic peptide receptor A,natriuretic peptide receptor A	natriuretic peptide receptor 1
NPR2	guanylate cyclase 2B,guanylyl cyclase B; acromesomelic dysplasia, Maroteaux type,atrionatriuretic peptide receptor B,natriuretic peptide receptor B; GUCY2B,ANPb,GC-B	natriuretic peptide receptor 2

NPR3	guanylate cyclase C; GUCY2B,FLJ14054; chromosome 5 open reading frame 23,atrionatriuretic peptide receptor C,natriuretic peptide receptor C/guanylate cyclase C (atrionatriuretic peptide receptor C),natriuretic peptide receptor C	natriuretic peptide receptor 3
NR1H2	liver X receptor-beta; NER,NER-I,RIP15,LXR-b,LXRb; ubiquitously-expressed nuclear receptor,nuclear receptor subfamily 1, group H, member 2	nuclear receptor subfamily 1 group H member 2
NR1H3	LXR-a,RLD-1,LXRa; nuclear receptor subfamily 1, group H, member 3; liver X receptor-alpha	nuclear receptor subfamily 1 group H member 3
NR1H4	FXR,RIP14,HRR1,HRR-1; nuclear receptor subfamily 1, group H, member 4; farnesoid X receptor,bile acid receptor	nuclear receptor subfamily 1 group H member 4
NR3C1	nuclear receptor subfamily 3, group C, member 1,nuclear receptor subfamily 3, group C, member 1 (glucocorticoid receptor); GR; glucocorticoid receptor	nuclear receptor subfamily 3 group C member 1
NRG1	NRG1 intronic transcript 2 (non-protein coding); HRG,NDF,GGF	neuregulin 1
NRG4	HRG4	neuregulin 4
NRIP3	chromosome 11 open reading frame 14	nuclear receptor interacting protein 3
NRXN1	KIAA0578,Hs.22998	neurexin 1
NRXN3	chromosome 14 open reading frame 60; KIAA0743	neurexin 3
NTRK2	neurotrophic tyrosine kinase, receptor, type 2; TRKB; BDNF/NT-3 growth factors receptor	neurotrophic receptor tyrosine kinase 2
OAS1	2',5'-oligoadenylate synthetase 1 (40-46 kD),2'-5'-oligoadenylate synthetase 1, 40/46kDa; OIASI,IFI-4; 2'-5' oligoadenylate synthase 1	2'-5'-oligoadenylate synthetase 1
OAS2	2'-5'-oligoadenylate synthetase 2 (69-71 kD),2'-5'-oligoadenylate synthetase 2, 69/71kDa	2'-5'-oligoadenylate synthetase 2
OAS3	2'-5'-oligoadenylate synthetase 3 (100 kD),2'-5'-oligoadenylate synthetase 3, 100kDa	2'-5'-oligoadenylate synthetase 3
OASL	2'-5'-oligoadenylate synthetase-like; TRIP14,p59OASL,OASL1	2'-5'-oligoadenylate synthetase like
OLFM4	OlfD,GW112,GC1	olfactomedin 4
OLR1	LOX-1,SCARE1,CLEC8A; oxidised low density lipoprotein (lectin-like) receptor 1	oxidized low density lipoprotein receptor 1
OSM	MGC20461	oncostatin M
OSMR	OSMRB,OSMRbeta; Oncostatin-M-specific receptor subunit beta	oncostatin M receptor
OXER1	5-oxo-ETE acid G-protein-coupled receptor 1,OXE receptor; GPCR,TG1019,GPR170	oxoeicosanoid receptor 1
OXGR1	2-oxoglutarate receptor 1,alpha-ketoglutarate receptor 1; P2RY15,P2Y15,aKGR; G protein-coupled receptor 80,oxoglutarate (alpha-ketoglutarate) receptor 1	oxoglutarate receptor 1
P2RX5	P2X5,LRH-1; purinergic receptor P2X, ligand-gated ion channel, 5	purinergic receptor P2X 5
P2RY12	purinergic receptor P2Y, G-protein coupled, 12; P2Y12,SP1999,HORK3	purinergic receptor P2Y12
PARP1	PARP,ARTD1; ADP-ribosyltransferase (NAD+; poly (ADP-ribose) polymerase),poly (ADP-ribose) polymerase family, member 1	poly(ADP-ribose) polymerase 1
PCNA		proliferating cell nuclear antigen
PDCD1	CD279,PD1,hSLE1,PD-1; systemic lupus erythematosus susceptibility 2	programmed cell death 1
PDCD1LG2	B7 dendritic cell molecule; PD-L2,Btdc,PDL2,bA574F11.2,CD273,B7-DC	programmed cell death 1 ligand 2
PDGFA	PDGF A-chain,platelet-derived growth factor alpha chain; platelet-derived growth factor alpha polypeptide; PDGF1,PDGF-A	platelet derived growth factor subunit A
PDGFB	oncogene SIS,becaplermin; platelet-derived growth factor beta polypeptide (simian sarcoma viral (v-sis) oncogene homolog),platelet-derived growth factor beta polypeptide; SSV	platelet derived growth factor subunit B
PDGFC	SCDGF,fallotein	platelet derived growth factor C
PDGFD	SCDGF-B,MSTP036,IEGF; spinal cord derived growth factor B	platelet derived growth factor D

PDGFRA	CD140a,PDGFR2,GAS9; platelet-derived growth factor receptor, alpha polypeptide	platelet derived growth factor receptor alpha
PDGFRB	JTK12,CD140b,PDGFR1; platelet-derived growth factor receptor, beta polypeptide	platelet derived growth factor receptor beta
PECAM1	CD31; CD31 antigen; platelet/endothelial cell adhesion molecule 1	platelet and endothelial cell adhesion molecule 1
PF4	SCYB4,CXCL4; chemokine (C-X-C motif) ligand 4	platelet factor 4
PGF	placenta growth factor; PlGF-2,SHGC-10760,D12S1900,PIGF,PLGF,PlGF; placental growth factor-like,placental growth factor, vascular endothelial growth factor-related protein	placental growth factor
PGR	PR,NR3C3	progesterone receptor
PHLDA2	IPL,BWR1C,HLDA2; tumor suppressing subtransferable candidate 3,pleckstrin homology-like domain, family A, member 2	pleckstrin homology like domain family A member 2
PIGR		polymeric immunoglobulin receptor
PLA2R1	PLA2G1R,PLA2IR,PLA2-R,CLEC13C; phospholipase A2 receptor 1, 180kD,phospholipase A2 receptor 1, 180kDa	phospholipase A2 receptor 1
PLAC8	placenta specific 8; onzin,C15	placenta associated 8
PNOC	nocistatin,orphanin FQ; PPNOC,N/OFQ,NOP	prepronociceptin
POU5F1	POU domain class 5, transcription factor 1; OCT3,Oct4,MGC22487	POU class 5 homeobox 1
PPARA	hPPAR,NR1C1; peroxisome proliferative activated receptor, alpha,peroxisome proliferator-activated receptor alpha	peroxisome proliferator activated receptor alpha
PPARD	NUC1,NUCII,FAAR,NR1C2; peroxisome proliferative activated receptor, delta,peroxisome proliferator-activated receptor delta	peroxisome proliferator activated receptor delta
PPARG	PPARG1,PPARG2,NR1C3,PPARgamma; peroxisome proliferative activated receptor, gamma,peroxisome proliferator-activated receptor gamma	peroxisome proliferator activated receptor gamma
PPBP	SCYB7,TGB,NAP-2-L1,LA-PF4,MDGF,LDGF,Beta-TG,CTAP3,CXCL7,PBP,b-TG1,TGB1,CTAPIII,NAP-2; platelet basic protein,beta-thromboglobulin,connective tissue-activating peptide III,neutrophil-activating peptide-2,chemokine (C-X-C motif) ligand 7	pro-platelet basic protein
PRF1	Perforin,perforin 1 (preforming protein); PFP,P1,HPLH2; perforin 1 (pore forming protein)	perforin 1
PROK2	PK2,BV8,MIT1,KAL4; protein Bv8 homolog	prokineticin 2
PROKR1	PKR1,ZAQ,GPR73a; G protein-coupled receptor 73	prokineticin receptor 1
PRSS2	trypsin 2; TRY2; protease, serine 2	serine protease 2
PSAP	sphingolipid activator protein-1,sphingolipid activator protein-2; variant Gaucher disease and variant metachromatic leukodystrophy,saposin-A,saposin-B,saposin-C,saposin-D	prosaposin
PSCA		prostate stem cell antigen
PTGDR2	CRTH2,CD294,DP2; G protein-coupled receptor 44; chemoattractant receptor homologous molecule expressed on T helper type 2 cells	prostaglandin D2 receptor 2
PTGDS	PGDS,L-PGDS; lipocalin-type prostaglandin D synthase; prostaglandin D2 synthase (21kD, brain),prostaglandin D2 synthase 21kDa (brain)	prostaglandin D2 synthase
PTGES	microsomal glutathione S-transferase 1-like 1,tumor protein p53 inducible protein 12,p53-induced gene 12,microsomal prostaglandin E synthase-1,glutathione S-transferase 1-like 1,MGST1-like 1; MGST-IV,PIG12,MGST1-L1,TP53I12	prostaglandin E synthase
PTGES2	chromosome 9 open reading frame 15; FLJ14038	prostaglandin E synthase 2
PTGES3	p23,TEBP,cPGES; prostaglandin E synthase 3 (cytosolic)	prostaglandin E synthase 3
PTGIS	PGIS,CYP8A1; prostaglandin I2 (prostacyclin) synthase; cytochrome P450, family 8, subfamily A, polypeptide 1,prostacyclin synthase	prostaglandin I2 synthase
PTGS1	COX1,PGHS-1,PTGHS; prostaglandin-endoperoxide synthase 1 (prostaglandin G/H synthase and cyclooxygenase); cyclooxygenase-1	prostaglandin-endoperoxide synthase 1

PTGS2	COX2; prostaglandin-endoperoxide synthase 2 (prostaglandin G/H synthase and cyclooxygenase); prostaglandin G/H synthase 2,cyclooxygenase 2	prostaglandin-endoperoxide synthase 2
PTHLH	osteostatin,parathyroid hormone-like hormone preproprotein,parathyroid hormone-related protein preproprotein; PTHRP,HHM,PLP,PTHR	parathyroid hormone like hormone
PTK2	FAK,FADK,FAK1,PPP1R71; protein phosphatase 1, regulatory subunit 71; PTK2 protein tyrosine kinase 2	protein tyrosine kinase 2
PTK6	BRK; PTK6 protein tyrosine kinase 6	protein tyrosine kinase 6
PTPRC	LCA,T200,GP180	protein tyrosine phosphatase receptor type C
PTPRCAP	LPAP,CD45-AP	protein tyrosine phosphatase receptor type C associated protein
PTTG1	PTTG,HPTTG,EAP1,securin; pituitary tumor-transforming 1; ESP1-associated protein 1,tumor-transforming protein 1	PTTG1 regulator of sister chromatid separation, securin
QRFPR	peptide P518 receptor; G protein-coupled receptor 103	pyroglutamylated RFamide peptide receptor
RAC1	TC-25,p21-Rac1,Rac-1; ras-related C3 botulinum toxin substrate 1 (rho family, small GTP binding protein Rac1)	Rac family small GTPase 1
RAC2	ras-related C3 botulinum toxin substrate 2 (rho family, small GTP binding protein Rac2); EN-7	Rac family small GTPase 2
RAD51	HsRad51,HsT16930,BRCC5,FANCR; BRCA1/BRCA2-containing complex, subunit 5; RAD51 (S. cerevisiae) homolog (E coli RecA homolog),RAD51 homolog (RecA homolog, E. coli) (S. cerevisiae),RAD51 homolog (S. cerevisiae)	RAD51 recombinase
RAMP1		receptor activity modifying protein 1
RAMP2		receptor activity modifying protein 2
RAMP3		receptor activity modifying protein 3
RARA	retinoic acid receptor, alpha; RAR,NR1B1	retinoic acid receptor alpha
RARB	HAP,NR1B2,RRB2; retinoic acid receptor, beta	retinoic acid receptor beta
RARG	RARC,NR1B3; retinoic acid receptor, gamma	retinoic acid receptor gamma
RARRES1	retinoic acid receptor responder (tazarotene induced) 1; TIG1,LXNL; latexin-like	retinoic acid receptor responder 1
RARRES2	retinoic acid receptor responder (tazarotene induced) 2; chemerin; TIG2,HP10433	retinoic acid receptor responder 2
RB1	prepro-retinoblastoma-associated protein,protein phosphatase 1, regulatory subunit 130; osteosarcoma,retinoblastoma 1; RB,PPP1R130	RB transcriptional corepressor 1
RBPJ	recombining binding protein suppressor of hairless (Drosophila); SUH,IGKJRB,RBPJK,KBF2,RBP-J,CBF1; suppressor of hairless homolog (Drosophila)	recombination signal binding protein for immunoglobulin kappa J region
REG1A	regenerating islet-derived 1 alpha (pancreatic stone protein, pancreatic thread protein); pancreatic stone protein,pancreatic thread protein; PSP,PTP,PSPS,PSPS1	regenerating family member 1 alpha
RELA	nuclear factor of kappa light polypeptide gene enhancer in B-cells 3,v-rel avian reticuloendotheliosis viral oncogene homolog A; p65	RELA proto-oncogene, NF-kB subunit
RELT	receptor expressed in lymphoid tissues; tumor necrosis factor receptor superfamily, member 19-like,RELT tumor necrosis factor receptor; FLJ14993	RELT TNF receptor
RGCC	response gene to complement 32; chromosome 13 open reading frame 15; bA157L14.2,RGC-32,RGC32	regulator of cell cycle
RGS1	1R20,IR20,BL34; regulator of G-protein signalling 1,regulator of G-protein signaling 1	regulator of G protein signaling 1
RGS2	regulator of G-protein signalling 2, 24kD,regulator of G-protein signalling 2, 24kDa,regulator of G-protein signaling 2, 24kDa,regulator of G-protein signaling 2	regulator of G protein signaling 2
RGS5	regulator of G-protein signalling 5,regulator of G-protein signaling 5	regulator of G protein signaling 5
RNF43	FLJ20315,DKFZp781H0392,URCC	ring finger protein 43
ROR1		receptor tyrosine kinase like orphan receptor 1
RORA	RAR-related orphan receptor A; RZRA,ROR1,ROR2,ROR3,NR1F1	RAR related orphan receptor A
RPL21	L21,FLJ27458,MGC71252,MGC104274,MGC104275,DKFZp686C0610 1; 60S ribosomal protein L21	ribosomal protein L21
RPL22	EAP,L22	ribosomal protein L22
RPL32	L32	ribosomal protein L32

RPL34	L34	ribosomal protein L34
RPL37	L37; 60S ribosomal protein L37a	ribosomal protein L37
RPS4Y1	ribosomal protein S4Y,40S ribosomal protein S4, Y; MGC5070,MGC119100,S4; ribosomal protein S4, Y-linked	ribosomal protein S4 Y-linked 1
RSPO1	R-spondin homolog (Xenopus laevis); FLJ40906,RSPONDIN	R-spondin 1
RSPO2	R-spondin 2 homolog (Xenopus laevis); MGC35555	R-spondin 2
RSPO3	FLJ14440; thrombospondin, type I, domain containing 2,R-spondin 3 homolog (Xenopus laevis)	R-spondin 3
RUNX3	AML2,PEBP2A3; runt-related transcription factor 3,runt related transcription factor 3	RUNX family transcription factor 3
RXRA	retinoid X receptor, alpha; nuclear receptor subfamily 2 group B member 1; NR2B1	retinoid X receptor alpha
RXRB	nuclear receptor subfamily 2 group B member 2; NR2B2,H-2RIIBP,RCoR-1; retinoid X receptor, beta	retinoid X receptor beta
RYK	JTK5A protein tyrosine kinase,RYK receptor-like tyrosine kinase; D3S3195,RYK1,JTK5	receptor like tyrosine kinase
S100A10	S100 calcium-binding protein A10 (annexin II ligand, calpactin I, light polypeptide (p11)); P11,42C,CLP11; annexin II tetramer (AIIt) p11 subunit,calpactin I	S100 calcium binding protein A10
S100A2	S100 calcium-binding protein A2; CAN19	S100 calcium binding protein A2
S100A4	P9KA,18A2,PEL98,42A,FSP1; fibroblast-specific protein-1,calvasculin,murine placental homolog; metastasin 1,calcium placental protein,S100 calcium-binding protein A4 (calcium protein, calvasculin, metastasin, murine placental homolog)	S100 calcium binding protein A4
S100A6	2A9,PRA,CABP; calcyclin,S100 calcium-binding protein A6 (calcyclin)	S100 calcium binding protein A6
S100A8	P8,MRP8,60B8AG,CGLA; calgranulin A,S100 calcium-binding protein A8 (calgranulin A)	S100 calcium binding protein A8
S100A9	calgranulin B,S100 calcium-binding protein A9 (calgranulin B); P14,MIF,NIF,LIAG,MRP14,MAC387,60B8AG,CGLB	S100 calcium binding protein A9
S100B	S100beta; S100 calcium binding protein, beta (neural)	S100 calcium binding protein B
S100P	S100 calcium-binding protein P	S100 calcium binding protein P
SAA1	PIG4,TP53I4	serum amyloid A1
SAA2		serum amyloid A2
SAT1	spermidine/spermine N1-acetyltransferase; diamine N-acetyltransferase 1; SSAT	spermidine/spermine N1-acetyltransferase 1
SCG5	secretory granule, neuroendocrine protein 1 (7B2 protein); prohormone convertase chaperone; 7B2,SgV	secretogranin V
SCGB3A1	UGRP2,HIN-1,HIN1,LU105,PnSP-2; cytokine high in normal-1,pneumo secretory protein 2	secretoglobin family 3A member 1
SEC23A	Sec23 (S. cerevisiae) homolog A,Sec23 homolog A (S. cerevisiae),Sec23 homolog A, COPII coat complex component	SEC23 homolog A, COPII coat complex component
SEC61G	SSS1; Sec61 gamma subunit	SEC61 translocon subunit gamma
SELENOP	SeP,SELP,SEPP; selenoprotein P, plasma, 1	selenoprotein P
SELL	LSEL,LAM1,LAM-1,hLHRc,Leu-8,Lyam-1,PLNHR,CD62L; lymphocyte adhesion molecule 1	selectin L
SELPLG	PSGL-1,CD162	selectin P ligand
SERPINA1	AAT,A1A,PI1,alpha-1-antitrypsin,A1AT,alpha1AT; serine (or cysteine) proteinase inhibitor, clade A (alpha-1 antiproteinase, antitrypsin), member 1,serpin peptidase inhibitor, clade A (alpha-1 antiproteinase, antitrypsin), member 1; protease inhibitor 1 (anti-elastase), alpha-1-antitrypsin	serpin family A member 1
SERPINA3	alpha-1-antichymotrypsin,serine (or cysteine) proteinase inhibitor, clade A (alpha-1 antiproteinase, antitrypsin), member 3,serpin peptidase inhibitor, clade A (alpha-1 antiproteinase, antitrypsin), member 3; ACT	serpin family A member 3
SERPINB5	serine (or cysteine) proteinase inhibitor, clade B (ovalbumin), member 5,serpin peptidase inhibitor, clade B (ovalbumin), member 5; protease inhibitor 5 (maspin); maspin	serpin family B member 5

SERPINH1	collagen binding protein 1; serine (or cysteine) proteinase inhibitor, clade H (heat shock protein 47), member 2,serine (or cysteine) proteinase inhibitor, clade H (heat shock protein 47), member 1, (collagen binding protein 1),serpin peptidase inhibitor, clade H (heat shock protein 47), member 1, (collagen binding protein 1); HSP47,colligen	serpin family H member 1
SFN	YWHAS; 14-3-3 sigma	stratifin
SIGIRR	single immunoglobulin domain IL1R1 related; single immunoglobulin and toll-interleukin 1 receptor (TIR) domain; TIR8,IL-1R8	single Ig and TIR domain containing
SLC2A1	DYT18,DYT9; human T-cell leukemia virus (I and II) receptor,choreoathetosis/spasticity, episodic (paroxysmal choreoathetosis/spasticity),solute carrier family 2 (facilitated glucose transporter), member 1	solute carrier family 2 member 1
SLC2A4	solute carrier family 2 (facilitated glucose transporter), member 4	solute carrier family 2 member 4
SLC40A1	solute carrier family 11 (proton-coupled divalent metal ion transporters), member 3,solute carrier family 40 (iron-regulated transporter), member 1; MTP1,IREG1,FPN1,HFE4; ferroportin 1	solute carrier family 40 member 1
SLPI	antileukoproteinase; secretory leukocyte protease inhibitor (antileukoproteinase); HUSI-I,ALK1,ALP,BLPI,HUSI,WAP4,WFDC4	secretory leukocyte peptidase inhibitor
SMAD2	MAD, mothers against decapentaplegic homolog 2 (Drosophila),SMAD, mothers against DPP homolog 2 (Drosophila); MADR2,JV18-1	SMAD family member 2
SMAD3	JV15-2,HsT17436; MAD, mothers against decapentaplegic homolog 3 (Drosophila),SMAD, mothers against DPP homolog 3 (Drosophila)	SMAD family member 3
SMAD4	DPC4; MAD, mothers against decapentaplegic homolog 4 (Drosophila),SMAD, mothers against DPP homolog 4 (Drosophila)	SMAD family member 4
SMARCB1	BAF47,Ini1,Snr1,hSNFS,Sfh1p,RDT,PPP1R144,SNF5; sucrose nonfermenting, yeast, homolog-like 1,integrase interactor 1,malignant rhabdoid tumor suppressor,protein phosphatase 1, regulatory subunit 144	SWI/SNF related, matrix associated, actin dependent regulator of chromatin, subfamily b, member 1
SMO	smoothened (Drosophila) homolog,smoothened homolog (Drosophila),smoothened, seven transmembrane spanning receptor,smoothened, frizzled family receptor; frizzled family member 11; FZD11	smoothened, frizzled class receptor
SNAI1	snail 1 (drosophila homolog), zinc finger protein,snail homolog 1 (Drosophila),snail family zinc finger 1; SNA,SLUGH2,SNAH,SNAIL1,SNAIL	snail family transcriptional repressor 1
SNAI2	slug homolog, zinc finger protein (chicken),snail homolog 2 (Drosophila),snail family zinc finger 2; SLUGH1,SNAIL2,SLUGH	snail family transcriptional repressor 2
SOD1	amyotrophic lateral sclerosis 1 (adult),superoxide dismutase 1, soluble; IPOA	superoxide dismutase 1
SOD2	gastric cancer–associated lncRNA 1; superoxide dismutase 2, mitochondrial; GClnc1	superoxide dismutase 2
SOSTDC1	DKFZp564D206,USAG1,DAND7; ectodin,wise	sclerostin domain containing 1
SOX2	SRY (sex determining region Y)-box 2,SRY-box 2	SRY-box transcription factor 2
SOX4	SRY (sex determining region Y)-box 4,SRY-box 4	SRY-box transcription factor 4
SOX9	campomelic dysplasia, autosomal sex-reversal,SRY (sex determining region Y)-box 9,SRY-box 9; SRA1	SRY-box transcription factor 9
SPARCL1	MAST9; SPARC-like 1 (mast9, hevin); hevin	SPARC like 1
SPINK1	serine protease inhibitor, Kazal type 1; Spink3,PCTT,PSTI,TATI	serine peptidase inhibitor Kazal type 1
SPOCK2	KIAA0275,testican-2; sparc/osteonectin, cwcv and kazal-like domains proteoglycan (testican) 2,SPARC/osteonectin, cwcv and kazal like domains proteoglycan 2	SPARC (osteonectin), cwcv and kazal like domains proteoglycan 2
SPP1	BSPI,ETA-1; osteopontin,bone sialoprotein I; early T-lymphocyte activation 1	secreted phosphoprotein 1
SPRY2	sprouty (Drosophila) homolog 2,sprouty homolog 2 (Drosophila); hSPRY2	sprouty RTK signaling antagonist 2

SPRY4	sprouty homolog 4 (Drosophila)	sprouty RTK signaling antagonist 4
SQSTM1	Paget disease of bone 3,oxidative stress induced like; autophagy receptor p62; p62,p60,p62B,A170	sequestosome 1
SRC	v-src avian sarcoma (Schmidt-Ruppin A-2) viral oncogene homolog; ASV,c-src	SRC proto-oncogene, non-receptor tyrosine kinase
SREBF1	SREBP1,bHLHd1,SREBP-1c,SREBP1a	sterol regulatory element binding transcription factor 1
SRGN	PPG; proteoglycan 1, secretory granule; serglycin proteoglycan	serglycin
SST	SMST,SST1; somatostatin-14,somatostatin-28,prepro-somatostatin	somatostatin
ST6GAL1	sialyltransferase 1 (beta-galactoside alpha-2,6-sialytransferase),ST6 beta-galactosamide alpha-2,6-sialyltranferase 1,ST6 N-acetylgalactosaminide alpha-2,6-sialyltransferase 1; ST6Gal I	ST6 beta-galactoside alpha-2,6-sialyltransferase 1
ST6GALNAC3	sialyltransferase 7 ((alpha-N-acetylneuraminyl-2,3-beta-galactosyl-1,3)-N-acetyl galactosaminide alpha-2,6-sialyltransferase) C,ST6 (alpha-N-acetyl-neuraminyl-2,3-beta-galactosyl-1,3)-N-acetylgalactosaminide alpha-2,6-sialyltransferase 3,ST6 GalNAc alpha-2,6-sialyltransferase 3; ST6GALNAC III	ST6 N-acetylgalactosaminide alpha-2,6-sialyltransferase 3
STAT1	STAT91,ISGF-3; transcription factor ISGF-3 components p91/p84; signal transducer and activator of transcription 1, 91kD,signal transducer and activator of transcription 1, 91kDa	signal transducer and activator of transcription 1
STAT3	signal transducer and activator of transcription 3 (acute-phase response factor); APRF	signal transducer and activator of transcription 3
STAT4		signal transducer and activator of transcription 4
STAT5A	MGF	signal transducer and activator of transcription 5A
STAT5B		signal transducer and activator of transcription 5B
STAT6	D12S1644,IL-4-STAT; signal transducer and activator of transcription 6, interleukin-4 induced	signal transducer and activator of transcription 6
STMN1	SMN,OP18,PR22,PP19,PP17,Lag,FLJ32206; chromosome 1 open reading frame 215,stathmin 1/oncoprotein 18; oncoprotein 18	stathmin 1
SUCNR1	G protein-coupled receptor 91	succinate receptor 1
SYK	spleen tyrosine kinase	spleen associated tyrosine kinase
TACSTD2	membrane component chromosome 1 surface marker 1; TROP2,GA733-1,EGP-1; epithelial glycoprotein-1,trophoblast cell surface antigen 2	tumor associated calcium signal transducer 2
TAGLN	SM22-alpha,transgelin variant 2; SM22,WS3-10,TAGLN1,SMCC,DKFZp686P11128	transgelin
TAP1	transporter 1, ATP-binding cassette, sub-family B (MDR/TAP); PSF1,RING4,D6S114E	transporter 1, ATP binding cassette subfamily B member
TAP2	transporter 2, ATP-binding cassette, sub-family B (MDR/TAP); PSF2,RING11,D6S217E	transporter 2, ATP binding cassette subfamily B member
TBX21	T-box 21; TBLYM,T-bet	T-box transcription factor 21
TCL1A	T cell leukemia/lymphoma 1A; TCL1	TCL1 family AKT coactivator A
TEK	angiopoietin-1 receptor; TIE2,TIE-2,VMCM1,CD202b; venous malformations, multiple cutaneous and mucosal,TEK tyrosine kinase, endothelial	TEK receptor tyrosine kinase
TFEB	TCFEB,bHLHe35	transcription factor EB
TGFB1	CED,TGFbeta; Camurati-Engelmann disease,prepro-transforming growth factor beta-1,Diaphyseal dysplasia 1, progressive; transforming growth factor, beta 1	transforming growth factor beta 1
TGFB2	transforming growth factor, beta 2; prepro-transforming growth factor beta-2	transforming growth factor beta 2
TGFB3	arrhythmogenic right ventricular dysplasia 1,transforming growth factor, beta 3; prepro-transforming growth factor beta-3	transforming growth factor beta 3
TGFBR1	activin A receptor type II-like kinase, 53kDa; multiple self-healing squamous epithelioma,transforming growth factor beta receptor I; ALK-5,ACVRLK4,ALK5,TBRI,TBR-i	transforming growth factor beta receptor 1
TGFBR2	transforming growth factor, beta receptor II (70/80kDa),transforming growth factor beta receptor II; TBRII,TBR-ii	transforming growth factor beta receptor 2

THBS1	TSP1,THBS,TSP,THBS-1,TSP-1; thrombospondin-1p180	thrombospondin 1
THBS2	TSP2	thrombospondin 2
TIE1	tyrosine kinase with immunoglobulin and epidermal growth factor homology domains 1,tyrosine kinase with immunoglobulin-like and EGF-like domains 1; JTK14	tyrosine kinase with immunoglobulin like and EGF like domains 1
TIGIT	VSIG9, VSTM3; FLJ39873,DKFZp667A205	T cell immunoreceptor with Ig and ITIM domains
TIMP1	tissue inhibitor of metalloproteinase 1 (erythroid potentiating activity, collagenase inhibitor); EPO	TIMP metallopeptidase inhibitor 1
TLR1	rsc786,KIAA0012,CD281; toll-like receptor 1	toll like receptor 1
TLR2	toll-like receptor 2; TIL4,CD282	toll like receptor 2
TLR3	CD283; toll-like receptor 3	toll like receptor 3
TLR4	toll-like receptor 4; hToll,CD284,TLR-4,ARMD10	toll like receptor 4
TLR5	systemic lupus erythematosus susceptibility 1,toll-like receptor 5; Toll/interleukin-1 receptor-like protein 3; TIL3,FLJ10052,MGC126430,MGC126431	toll like receptor 5
TLR7	toll-like receptor 7	toll like receptor 7
TLR8	CD288; toll-like receptor 8	toll like receptor 8
TM4SF1	transmembrane 4 superfamily member 1; L6	transmembrane 4 L six family member 1
TNF	TNF superfamily, member 2; tumor necrosis factor (TNF superfamily, member 2); TNFSF2,DIF,TNF-alpha	tumor necrosis factor
TNFAIP6	tumor necrosis factor, alpha-induced protein 6; TSG6,TSG-6	TNF alpha induced protein 6
TNFRSF10A	tumor necrosis factor receptor superfamily, member 10a; DR4,Apo2,TRAILR-1,CD261,TRAILR1	TNF receptor superfamily member 10a
TNFRSF10B	DR5,KILLER,TRICK2A,TRAIL-R2,TRICKB,CD262,TRAILR2; tumor necrosis factor receptor superfamily, member 10b	TNF receptor superfamily member 10b
TNFRSF10D	DcR2,TRUNDD,TRAILR4,CD264; tumor necrosis factor receptor superfamily, member 10d, decoy with truncated death domain	TNF receptor superfamily member 10d
TNFRSF11A	osteoclast differentiation factor receptor,receptor activator of nuclear factor kappa B,familial expansile osteolysis,TRANCE receptor; RANK,CD265,FEO,ODFR,TRANCE-R; tumor necrosis factor receptor superfamily, member 11a, activator of NFKB,Paget disease of bone 2,loss of heterozygosity, 18, chromosomal region 1,tumor necrosis factor receptor superfamily, member 11a, NFKB activator	TNF receptor superfamily member 11a
TNFRSF11B	osteoclastogenesis inhibitory factor; osteoprotegerin,tumor necrosis factor receptor superfamily, member 11b; OCIF,TR1	TNF receptor superfamily member 11b
TNFRSF12A	FN14,TweakR,CD266; tumor necrosis factor receptor superfamily, member 12A	TNF receptor superfamily member 12A
TNFRSF13B	TACI,CD267,IGAD2; tumor necrosis factor receptor superfamily, member 13B	TNF receptor superfamily member 13B
TNFRSF14	herpesvirus entry mediator; HVEM,ATAR,TR2,LIGHTR,HVEA,CD270; tumor necrosis factor receptor superfamily, member 14 (herpesvirus entry mediator),tumor necrosis factor receptor superfamily, member 14	TNF receptor superfamily member 14
TNFRSF17	BCM,CD269,TNFRSF13A; tumor necrosis factor receptor superfamily, member 17	TNF receptor superfamily member 17
TNFRSF18	tumor necrosis factor receptor superfamily, member 18; AITR,GITR,CD357	TNF receptor superfamily member 18
TNFRSF19	toxicity and JNK inducer; TAJ-alpha,TROY,TAJ,TRADE; tumor necrosis factor receptor superfamily, member 19	TNF receptor superfamily member 19
TNFRSF1A	tumor necrosis factor receptor superfamily, member 1A; TNF-R,TNFAR,TNFR60,TNF-R-I,CD120a,TNF-R55	TNF receptor superfamily member 1A
TNFRSF1B	tumor necrosis factor receptor superfamily, member 1B; TNFBR,TNFR80,TNF-R75,TNF-R-II,p75,CD120b	TNF receptor superfamily member 1B
TNFRSF21	death receptor 6; DR6,CD358; tumor necrosis factor receptor superfamily, member 21	TNF receptor superfamily member 21
TNFRSF4	tumor necrosis factor receptor superfamily, member 4; ACT35,OX40,CD134	TNF receptor superfamily member 4
TNFRSF9	tumor necrosis factor receptor superfamily, member 9; CD137,4-1BB	TNF receptor superfamily member 9

TNFSF10	TRAIL,Apo-2L,TL2,CD253; tumor necrosis factor (ligand) superfamily, member 10	TNF superfamily member 10
TNFSF12	tumor necrosis factor (ligand) superfamily, member 12; TWEAK,DR3LG,APO3L	TNF superfamily member 12
TNFSF13B	B-cell-activating factor,TNF and ApoL-related leukocyte expressed ligand 1,TNF homolog that activates apoptosis,B-lymphocyte stimulator; tumor necrosis factor (ligand) superfamily, member 13b; BAFF,THANK,BLYS,TALL-1,TALL1,CD257	TNF superfamily member 13b
TNFSF14	tumor necrosis factor (ligand) superfamily, member 14; LIGHT,LTg,HVEM-L,CD258	TNF superfamily member 14
TNFSF15	tumor necrosis factor (ligand) superfamily, member 15; TL1,VEGI,TL1A,VEGI192A,MGC129934,MGC129935; vascular endothelial cell growth inhibitor,TNF superfamily ligand TL1A,TNF ligand-related molecule 1,vascular endothelial growth inhibitor-192A	TNF superfamily member 15
TNFSF18	AITRL,TL6,hGITRL; tumor necrosis factor (ligand) superfamily, member 18	TNF superfamily member 18
TNFSF4	tax-transcriptionally activated glycoprotein 1, 34kD,tumor necrosis factor (ligand) superfamily, member 4; OX-40L,gp34,CD252	TNF superfamily member 4
TNFSF8	tumor necrosis factor (ligand) superfamily, member 8; CD153	TNF superfamily member 8
TNFSF9	tumor necrosis factor (ligand) superfamily, member 9; receptor 4-1BB ligand,homolog of mouse 4-1BB-L; 4-1BB-L,4-1BBL	TNF superfamily member 9
TOP2A	topoisomerase (DNA) II alpha 170kDa; TOP2alpha,TOPIIA	DNA topoisomerase II alpha
TOX	KIAA0808,TOX1	thymocyte selection associated high mobility group box
TP53	p53,LFS1; Li-Fraumeni syndrome	tumor protein p53
TPM1	chromosome 15 open reading frame 13,cardiomyopathy, hypertrophic 3,tropomyosin 1 (alpha)	tropomyosin 1
TPM2	DA1,NEM4; arthrogryposis multiplex congenital, distal, type 1,tropomyosin 2 (beta); nemaline myopathy type 4	tropomyosin 2
TPSAB1	tryptase alpha II,tryptase beta I,tryptase-I,tryptase-II,tryptase-III; tryptase beta 1	tryptase alpha/beta 1
TPSB2	tryptase beta 2 (gene/pseudogene); tryptase beta II,tryptase beta III	tryptase beta 2
TSC22D1	transforming growth factor beta 1 induced transcript 4,TSC22 domain family, member 1; TSC22,MGC17597	TSC22 domain family member 1
TSHZ2	ZABC2,OVC10-2,TSH2; chromosome 20 open reading frame 17,zinc finger protein 218,teashirt family zinc finger 2	teashirt zinc finger homeobox 2
TSLP		thymic stromal lymphopoietin
TTR	prealbumin, amyloidosis type I,carpal tunnel syndrome 1; HsT2651,CTS	transthyretin
TUBB	OK/SW-cl.56,MGC16435,M40,Tubb5; class I beta-tubulin,beta1-tubulin; tubulin, beta polypeptide,tubulin, beta,tubulin, beta class I	tubulin beta class I
TUBB4B	Beta2; class IVb beta-tubulin; tubulin, beta 2C	tubulin beta 4B class IVb
TWIST1	SCS,H-twist,BPES2,bHLHa38,CRS1; blepharophimosis, epicanthus inversus and ptosis 3,acrocephalosyndactyly 3,twist homolog 1 (Drosophila),twist basic helix-loop-helix transcription factor 1,craniosynostosis; Saethre-Chotzen syndrome	twist family bHLH transcription factor 1
TWIST2	twist homolog 2 (Drosophila),twist basic helix-loop-helix transcription factor 2; DERMO1,Dermo-1,bHLHa39	twist family bHLH transcription factor 2
TXK	TKL,PSCTK5,BTKL,RLK; PTK4 protein tyrosine kinase 4	TXK tyrosine kinase
TYK2	JTK1	tyrosine kinase 2
TYMS	Tsase,TMS,HsT422	thymidylate synthetase
TYROBP	polycystic lipomembranous osteodysplasia with sclerosing leukoencephalopathy,TYRO protein tyrosine kinase binding protein; DAP12,PLO-SL,KARAP; killer activating receptor associated protein,DNAX-activation protein 12,DNAX adaptor protein 12	transmembrane immune signaling adaptor TYROBP
UBE2C	UBCH10; ubiquitin-conjugating enzyme E2C	ubiquitin conjugating enzyme E2 C

UCP1	SLC25A7; uncoupling protein 1 (mitochondrial, proton carrier)	uncoupling protein 1
UPK3A	uroplakin 3	uroplakin 3A
VCAM1	CD106	vascular cell adhesion molecule 1
VCAN	chondroitin sulfate proteoglycan 2; PG-M; versican proteoglycan	versican
VEGFA	VEGF-A,VPF; vascular endothelial growth factor	vascular endothelial growth factor A
VEGFB	VEGFL	vascular endothelial growth factor B
VEGFC	vascular endothelial growth factor-related protein; VRP,VEGF-C	vascular endothelial growth factor C
VEGFD	c-fos induced growth factor (vascular endothelial growth factor D); VEGF-D	vascular endothelial growth factor D
VHL	von Hippel-Lindau syndrome,von Hippel-Lindau tumor suppressor, E3 ubiquitin protein ligase; VHL1	von Hippel-Lindau tumor suppressor
VIM		vimentin
VPREB3	8HS20; pre-B lymphocyte 3	V-set pre-B cell surrogate light chain 3
VSIR	SISP1,GI24,B7-H5,B7H5,VISTA,PD-1H,Dies1; stress induced secreted protein 1,V-domain Ig suppressor of T cell activation,PDCD1 homolog; chromosome 10 open reading frame 54	V-set immunoregulatory receptor
VTN	vitronectin (serum spreading factor, somatomedin B, complement S-protein); VN; serum spreading factor,somatomedin B,complement S-protein	vitronectin
VWF		von Willebrand factor
WIF1		WNT inhibitory factor 1
WNT10B	wingless-type MMTV integration site family, member 10B; WNT-12,SHFM6	Wnt family member 10B
WNT11	wingless-type MMTV integration site family, member 11	Wnt family member 11
WNT2	IRP; wingless-type MMTV integration site family member 2; secreted growth factor	Wnt family member 2
WNT2B	XWNT2; wingless-type MMTV integration site family, member 2B; XWNT2, Xenopus, homolog of,wingless-type MMTV integration site family, member 13	Wnt family member 2B
WNT3	wingless-type MMTV integration site family, member 3; MGC131950,MGC138321,MGC138323; WNT-3 proto-oncogene protein	Wnt family member 3
WNT5A	hWNT5A; WNT-5A protein; wingless-type MMTV integration site family, member 5A	Wnt family member 5A
WNT5B	wingless-type MMTV integration site family, member 5B	Wnt family member 5B
WNT7A	wingless-type MMTV integration site family, member 7A; proto-oncogene Wnt7a protein; Wnt-7a	Wnt family member 7A
WNT7B	wingless-type MMTV integration site family, member 7B	Wnt family member 7B
WNT9A	wingless-type MMTV integration site family, member 14,wingless-type MMTV integration site family, member 9A	Wnt family member 9A
XBP1		X-box binding protein 1
XCL1	lymphotactin; small inducible cytokine subfamily C, member 1 (lymphotactin),chemokine (C motif) ligand 1; LPTN,ATAC,SCM-1a,SCM-1	X-C motif chemokine ligand 1
XCL2	small inducible cytokine subfamily C, member 2,chemokine (C motif) ligand 2; SCM-1b	X-C motif chemokine ligand 2
YBX3	cold-shock domain containing A1; dbpA,ZONAB,CSDA1; cold shock domain protein A	Y-box binding protein 3
YES1	Yes,c-yes,HsT441; v-yes-1 Yamaguchi sarcoma viral oncogene homolog 1	YES proto-oncogene 1, Src family tyrosine kinase
ZFP36	zinc finger protein 36, C3H type, homolog (mouse); tristetraprolin; RNF162A,TIS11,G0S24,TTP,NUP475	ZFP36 ring finger protein
